# Elevation, an emotion for prosocial contagion, is experienced more strongly by those with greater expectations of the cooperativeness of others

**DOI:** 10.1371/journal.pone.0226071

**Published:** 2019-12-04

**Authors:** Adam Maxwell Sparks, Daniel M. T. Fessler, Colin Holbrook

**Affiliations:** 1 Department of Anthropology, University of California Los Angeles, Los Angeles, California, United States of America; 2 Center for Behavior, Evolution and Culture, University of California Los Angeles, Los Angeles, California, United States of America; 3 Bedari Kindness Institute, University of California Los Angeles, Los Angeles, California, United States of America; 4 Department of Cognitive and Information Sciences, University of California Merced, Merced, California, United States of America; Institut Francais des Sciences et Technologies des Transports de l'Amenagement et des Reseaux, FRANCE

## Abstract

A unique emotion, elevation, is thought to underlie prosocial contagion, a process whereby witnessing a prosocial act leads to acting prosocially. Individuals differ in their propensity to experience elevation, and thus their proneness to prosocial contagion, but little is known about the causes of such variation. We introduce an adaptationist model wherein elevation marks immediate circumstances in which generalized prosociality is advantageous, with this evaluation of circumstances hinging in part on prior expectations of others’ prosociality. In 15 studies, we add to evidence that elevation can reliably be elicited and mediates prosocial contagion. Importantly, we confirm a novel prediction–generated by our adaptationist account–that an idealistic attitude, which indexes others’ expected degree of prosociality, moderates the relationship between exposure to prosocial cues and experiencing elevation. We discuss how our findings inform both basic theorizing in the affective sciences and translational efforts to engineer a more harmonious world, and we offer future research directions to further test and extend our model.

## Introduction

Increasing the basic scientific understanding of psychological mechanisms underlying prosocial behavior is of theoretical importance across the social sciences, and has applied significance for engineering cooperative institutions [1]. Many emotions appear to play a role in prosocial behavior [2–6], among them *elevation*, the uplifting affect experienced upon witnessing others’ morally praiseworthy actions [7].

Here we present a novel adaptationist model wherein elevation functions to upregulate prosocial behavior in situations that are especially favorable for cooperative investments. The psychological mechanisms that regulate elevation must do so by interpreting when ambiguous social cues suggest that the present situation is ripe for investing in prosociality, where such interpretation is informed by prior social expectations. This leads to a novel empirical prediction, that individual differences in elevation response to similar social cues should be a function of differences in baseline expectations about the cooperativeness of others.

We then report 15 original empirical studies (*total N* = 8,118), the results of which replicate and extend several previously-reported elevation findings, and, importantly, also strongly support our novel prediction. Our open data set thus both adds confidence regarding previous findings that informed our theoretical model and offers novel evidence for a crucial feature of our model.

### A brief review of scholarship on elevation

Drawing heavily on prior work, especially two recent review papers in the field of positive psychology [8,9], but interpreting the empirical record through the lens of our model, we characterize elevation using four key features useful for differentiating among emotions [10,11]: (1) clusters of culturally-embedded folk affect terms used to describe the experience, (2) experienced physiological responses, (3) motivational/behavioral patterns, and (4) eliciting conditions.

Lay English speakers have no precise term for elevation, but “lifted up,” “inspired,” “moved,” “respect,” and “awe” are folk affect terms often used to describe the experience. Somatic symptoms of elevation include warmth in the chest, chills or goosebumps, a lump in the throat, and tears in the eyes. Elevation involves motivations to help others and be a better person, motives that appear to cause actual cooperative behavior [12–15]. The cooperative motives associated with elevation appear to be generalized, rather than directed at a specific target, as is the case with gratitude [16]. Self-report measures of elevation vary in their details, but typically involve Likert scale measures based on some or all of these first three characteristics of elevation: folk terms, somatic sensations, and prosocial motives. These three features are the basis of three corresponding subscales in the elevation measures we describe below. (We will argue that a fourth category of items commonly used in elevation scales, statements which capture general views of humanity, are better treated as measuring an *attitude* rather than part of the emotion itself.)

Our interpretation is that elevation is elicited by *exceptional prosocial behavior*. Prosocial behavior refers to one or more persons conferring a benefit on one or more other persons, at some immediate cost to the actor(s), i.e., cooperation. “Exceptional” implies that the witnessed prosociality is rare, extreme, spectacular, or in some other way violates the expectations of the witness, i.e., the set of stimuli that elicit elevation is some region within a continuum of prosocial behaviors from mundane to remarkable. Our perspective is somewhat different from that of other researchers. Both of the recent review papers define “witnessing moral beauty” as the eliciting condition of elevation, language that stems from Haidt’s [7] formal introduction of the construct of elevation to the modern scientific community as being an emotion elicited by “acts of human moral beauty or virtue,” a sketch which itself followed from Thomas Jefferson’s [17] musings about strong emotional responses to reading fictional “acts of charity or gratitude” that “deeply impress with beauty,” such as great acts of “fidelity” or “generosity.” Haidt elaborated that elicitors of elevation include “unexpected acts of human goodness, kindness, and compassion,” and especially charitable behavior. Clearly there is a great deal of overlap between the behavior sets marked by “exceptional cooperation” and “moral beauty,” and the same may be said for the notion of “moral heroism” [18]. Our interpretation of elevation’s elicitor set differs from the prevailing interpretation in the field of positive psychology by emphasizing the flow of costs and benefits between interacting parties rather than relying on moral and/or aesthetic judgments of these interactions. It is not our present goal to attempt a full analysis of the (in)compatibilities of these two models. Rather, we report and interpret supportive evidence for a prediction about individual differences in elevation that was facilitated by our theoretical linkage of elevation to theories regarding the evolution and maintenance of cooperative behavior, as we elaborate below.

### Elevation as part of an adaptive affective system for prosocial contagion

We can use the above sketch of the form of the elevation to infer its function; in the language of evolutionary biology, we can use information about the structure of the proximate mechanism to generate models of ultimate explanations for its existence. In turn, ultimate models can be used to generate hypotheses used to guide further empirical investigation of mechanisms [19,20]. Our general approach to modeling emotions as evolved biological mechanisms builds from Gervais and Fessler’s [11] Attitude-Scenario-Emotion framework, which we tailor to the current topic based on a functional analysis of the cooperative inputs and outputs of elevation. The resulting model generates hypotheses about the attitudes that are likely to regulate this emotion, yielding the testable prediction, investigated in the empirical portion of this paper, that individual differences in elevation experiences stem from individual’s prior expectations regarding the cooperativeness of other people.

### General adaptationist model of affective systems

The Attitude-Scenario-Emotion model [11] is a novel synthetic framework, developed with a focus on emotions that regulate dyadic social relationships. To our knowledge, the work reported here is the first extensive use of this framework in empirical investigation of a specific emotion, and, as we later elaborate, the first extension of the model beyond dyads.

Building on much prior work, Gervais and Fessler [11] distinguish between emotions and attitudes (and also sentiments, which we address in the General Discussion). *Emotions* are fast-acting and short-lived responses to events that reorganize psychological and physiological systems to quickly respond to a *scenario*, the challenges or opportunities indexed by particular sets of stimuli. Social scenarios require subjective interpretation in light of one’s specific circumstances. The appropriate emotional responses depend on (among other things) the people involved, and, more specifically, their expected behaviors and the value of those behaviors for the would-be responder. Such expectations are encoded as *attitudes*, and thus, in the Attitude-Scenario-Emotion framework, differences in attitudes can cause differences in interpretation of scenarios, which in turn cause differences in emotion and behavior. We will use this hypothesized general structure to predict and explain individual variation specific to elevation experiences.

### Functional theories of prosocial behavior

The critical outputs of the elevation mechanism are prosocial motivation and subsequent behavior. Accordingly, our functionalist approach to explaining a mechanism that causes cooperation draws on extensive literature addressing ultimate explanations of how individuals can enhance biological fitness through cooperation. Such explanations variously invoke the functional logic of kinship, reciprocity, mutualism, vested interest, and costly signaling, all in the context of competition for social partners within biological markets [21,22].

Critically, with the exception of kin-based altruism, all known individually-adaptive explanations of cooperation require that an actor’s investment in costly prosocial behavior leads to a subsequent payoff via benefits received from others. The fitness-profitability of prosociality is thus contingent on others’ actions; correspondingly, humans appear capable of behavior that is situationally contingent on expectations of others’ prosociality. In experimental paradigms in which participants face real-stakes cooperative dilemmas, individual cooperativeness is influenced by the apparent cooperativeness of other players [23–34]. Even experimental exposure to information about the apparent cooperativeness of distant others has similar effects [35–37]. Expanding further, field studies suggest that a variety of cues about prevailing levels of local prosociality contagiously inform a host of social behaviors [38–42].

### Our model of elevation and idealism

Against this backdrop, we posit that elevation is a mechanism that motivates adaptive upregulation of prosocial behavior following the interpretation of social cues as indicating that the current situation is ripe for cooperative investment, i.e., this emotion is a mechanism for adaptive *prosocial contagion*. (It is not our claim that elevation is the only such emotion; in the General Discussion we compare elevation to other putative prosocial emotions.)

Consider the case of witnessing an exceptionally prosocial exemplar, a common experimental method for eliciting elevation. We essentially agree with Algoe & Haidt’s [16] interpretation that, in such situations, “other-praising emotions” like elevation have prestige-biased learning functions [43], and we specifically connect this to the functional logic of cooperation as follows: The exceptionally prosocial exemplar provides a cue that the present environment is one in which prosociality may be especially rewarding, not only because the exemplar herself may be a good candidate to reciprocate [7], but also because her current prosociality implies that her past prosociality has been rewarded rather than exploited by third parties, indirectly suggesting that the local environment includes other cooperators and/or prosocial punishers who guard against antisocial behavior [24,44–46]. Thus, the individual who witnesses a prosocial exemplar may infer that she would likely benefit from behaving cooperatively too, and such benefits might derive from many people or pathways, explaining the diffuse (rather than targeted) prosocial motives noted by Algoe & Haidt [16]. (Note that the inferences we are describing are at a functional level; this need not imply that individuals are consciously aware of such inferences. Just as disgust might motivate someone to recoil from feces without conscious concern for pathogen-inflicted costs, elevation might motivate prosocial contagion without any conscious concern for cooperatively-delivered benefits.)

One pragmatic difficulty confronting a mechanism for adaptive prosocial contagion is that the social cues to be interpreted are inherently ambiguous, such that executing the above adaptive logic necessarily requires preliminary interpretive processing that produces such functional inferences as:

- The behavior I just witnessed was prosocial.
- The cooperative individual(s) are likely to benefit from their actions.
- I would benefit from similar acts.

Here we approach variation in the experience of elevation as being derived from individual differences that should make such inferences (whether implicit or explicit) more likely. When making such inferences, Type I errors are possible; the detection of an apparently benevolent actor is an imperfect cue to the future profitability of prosociality for the observer, especially for an observer new to the context. For example, a seeming act of kindness could, in fact, be bait in a trap for later exploitation. Given imperfect information, how then do observers determine how to condition current behavior on the informational value of the cue? In a Bayesian sense, the estimated likelihood that a given hypothesis is true (e.g., that this is a good time/place to behave prosocially) depends both on the immediate evidence and the prior estimate that the hypothesis is true. Thus, one’s prior expectations about how people tend to respond to prosocial behavior should regulate one’s interpretation of—and thus emotional and behavioral reactions to—an observed act of apparent extreme prosociality. (In the empirical work presented here, we measured participants’ prior expectations and their emotional and behavior responses, but did not measure their interpretations of the actions that they had witnessed, a decision reflecting the lack of a priori theoretical reasons for expecting that such functional inferences will necessarily be available to conscious mechanisms.)

In our model, these prior expectations regarding others’ prosociality are encoded in attitudes. In the dyadic framework introduced by Gervais and Fessler, an attitude towards an individual summarizes the attitude-holder’s expectations and valuations of that person, based on previous experiences. Attitudes can be characterized along a dimension we label “idealism-cynicism.” A more idealistic attitude towards someone indexes the expectation that the person tends to treat people prosocially; a more cynical (i.e., less idealistic) attitude towards someone corresponds to the expectation of more selfish behavior. An individual who has more idealistic prior attitudes towards the people involved in a social scenario should be more likely to interpret the witnessed behavior of the apparently prosocial exemplar as genuinely prosocial; more likely to expect cooperative responses to that behavior by others; and more likely to expect similar treatment herself in response to her own prosocial behavior. Conversely, a more cynical attitude towards the same individuals makes it less likely that the exemplar’s actions are interpreted as genuinely prosocial; less likely that other actors are expected to respond cooperatively to the original action; and less likely that the witness will assume that her own prosocial behavior would be rewarded. Accordingly, a witness’s idealism-cynicism towards the actors in a scenario (for simplicity, hereafter *idealism*) should moderate elevation and subsequent behavior in response to that scenario.

### One key empirical implication of theoretically distinguishing between elevation and idealism

In Gervais and Fessler’s Attitude-Scenario-Emotion framework, the relationship between attitudes and emotions is bidirectional. Attitudes summarize past experience to adaptively shape emotional responses to immediate circumstances, where such experience is both accrued by the individual and framed by predispositions that may have been formulated over deep evolutionary time (for example, a sex difference in the propensity to construe social relationships as, respectively, competitive or cooperative [47]); in turn, emotions provide input that updates the attitude in light of present experience. Because attitudes aggregate across many occurrences, with the exception of extraordinary events, a given emotional experience will make only an incremental contribution to the corresponding enduring attitude. Nevertheless, we can expect that information in attitudinal format will be part of the output of an emotion, as this is the medium through which Bayesian updating of attitudes occurs.

Given this framework, tentative support can be found in existing literature for our novel model of the relationship between idealistic attitudes and elevation. Elevation researchers commonly suggest that elevation results in feeling close to others, and self-report scales intended to measure the experience of elevation frequently include items such as “I feel optimistic about humanity.” In our perspective, feelings and cognitive assessments about others are attitudinal, rather than emotional. Thus, research to date appears to have merged the emotion of elevation, the underlying attitudinal dimension of idealism (directed towards “humanity” rather than a specific individual), and the feedback between the former and the latter.

Building on others’ extensive prior work, below we document our efforts to design and implement experimental methods that create elevation-mediated prosocial contagion, and to test the unique proposal that individual differences in the propensity to experience elevation, and subsequently upregulate prosocial motivations and behavior, importantly stem from variation in baseline expectations of others’ prosociality–that is, from individual differences in prior idealistic attitudes. Thus, the present empirical effort attempts to both replicate key effects that informed our novel model of elevation and test a novel prediction generated by this model.

**Fig 1:**
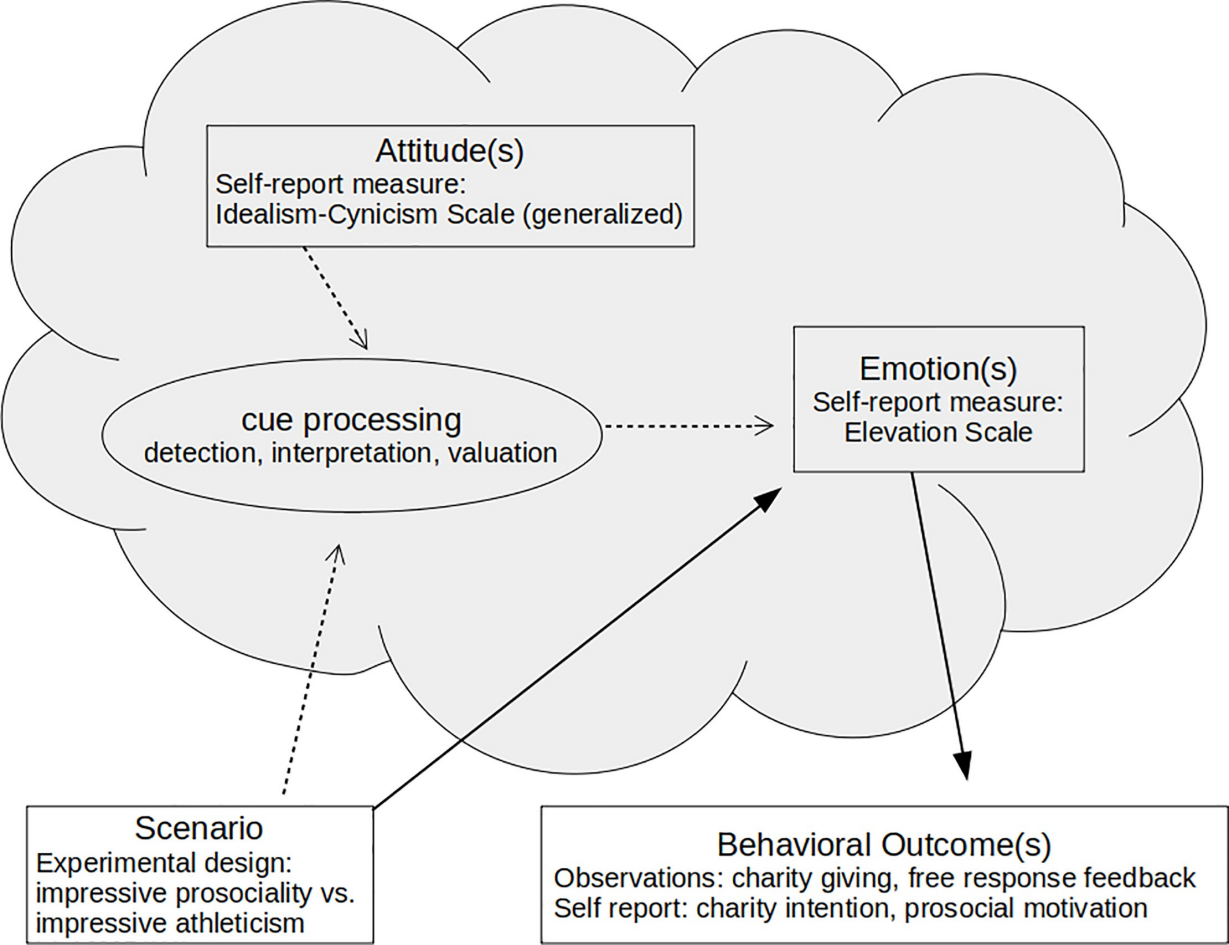
Structure of the elevation-idealism system. Hypothesized psychological processes are shown in grey. Processes measured in our methods are depicted in rectangles; a posited process not directly measured here is depicted as an oval. The conventional model of elevation (solid arrows) is of an emotional response to prosocial scenarios which causes prosocial behavioral responses. Our novel addition (dotted arrows), based on the Attitude-Scenario-Emotion framework, is that adaptive deployment of emotion requires interpretation of scenarios, a processing step which depends on previous experiences encoded as attitudes. Thus, attitudes should moderate the relationship between scenarios and emotions. Specifically, elevation should be regulated by idealism, an attitudinal dimension summarizing the expectation that (relevant) others tend to be cooperative and trustworthy. Additional causal arrows deserve investigation (see the General Discussion).

Fig 1 summarizes how the ASE model is applied to case of elevation, and how several of the model’s key concepts are operationalized in our methods.

## Methods

We have conducted multiple pre-registered, large-sample studies aimed at testing various aspects of our model. Our primary results here are meta-analyses of open data from all available studies that allow us to estimate the relationships between idealism, elevation, and behavior across varying types of scenarios (*K* = 15).

In all studies, participants completed a self-report scale intended to measure generalized idealism, then watched one or two videos and self-reported their elevation response to each. These data allow us to (1) assess how distributions of self-report emotional response outcomes differ by eliciting scenario (i.e., video type) and (2) test whether baseline idealism explains individual variation in elevation. In most studies, this was followed by one or more measures of real or hypothetical prosocial behavior, allowing an assessment of the relationship between emotion and behavior. As described and discussed below, and detailed in S1 Appendix, we refined our methods, measures, and stimuli across this series of studies and in several pilot efforts.

Table 1 summarizes the methods.

**Table 1:** Sample information.

Study	Starting n	Final n (all conditions)	(Athletic)	(Prosocial)	(Neutral)	Population
**study01**	526	499	-	240	-	MTurk workers
**study02**	521	494	262	232	-	MTurk workers
**study03**	602	569	276	293	569	MTurk workers
**study04**	211	197	90	107	-	Angelinos
**study05**	541	456	220	236	456	MTurk workers
**study06**	809	689	314	375	689	MTurk workers
**study07**	816	727	354	373	727	MTurk workers
**study08**	472	440	208	232	-	Angelinos
**study09**	454	431	228	203	-	Angelinos
**study10**	595	576	280	296	-	Angelinos
**study11**	224	184	85	99	-	MTurk workers
**study12**	302	257	128	129	-	MTurk workers
**study13**	1804	1616	271	276	1616	MTurk workers
**study14**	607	499	-	-	499	MTurk workers
**study15**	604	484	-	-	484	MTurk workers

Note that exposure to Athletic or Prosocial stimuli was implemented in between-subjects experimental designs, whereas the Neutral stimulus, when included in a study, was presented to all participants prior to the experimental exposure. See S1 Appendix for details.

### Participants and recruitment

Participants in field studies (*k* = 4) were recruited in public places in Los Angeles (mostly, but not exclusively, on or near the UCLA campus) and participated on the spot using tablet computers. Participants in all other studies (*k* = 11) were recruited via Amazon.com’s Mechanical Turk internet crowdsourcing platform (hereafter MTurk) to complete the study online. Online and field participants were recruited for “an [academic survey / research study] about feelings and memory”. Repeat participation across online studies was automatically precluded based on MTurk worker ID in most cases. Recruitment in field studies also attempted to exclude repeat participants; additionally, these studies generally targeted people who were alone in public, rather than with a group, although these were not strict requirements (we also conducted field studies, not reported here, in which pairs of individuals were specifically targeted for recruitment); all individual participants in field studies described here were removed from close proximity with other people at the time of participation. In our online studies, participants were randomly assigned to experimental condition; in field studies, conditions alternated across successive participants.

### Research ethics statement

All studies complied with ethical regulations and were approved by the UCLA Office of the Human Research Protection Program under protocol #14–001681. Informed consent was obtained from all participants.

### Video stimuli

Most of our studies involved an experimental contrast between the emotional and behavioral effects of exposure to, respectively, a prosocial video and an athletic video. In several online studies, participants watched a neutral video and recorded their elevation response prior to the aforementioned experimental intervention. In some studies, some or all participants saw only one of these three types of videos–such designs obviously do not allow direct experimental comparisons, but are included here in meta analyses estimating effects specific to a relevant video.

### Prosocial video

Our prosocial video consisted of a lightly edited version of *Unsung Hero*, a Thai television commercial. This 3-minute video, employed by other emotion researchers as well [48], depicts a young man engaging in a variety of helpful acts (e.g., giving money to a beggar, feeding a stray dog, helping a street vendor lift her cart); as the story progresses viewers witness positive consequences for those the protagonist helps, and recipients’ displays of gratitude and positive orientation toward him. Thus, this video contains numerous cues indicative of both the presence of a prosocial primary actor, and eventual rewards for his prosociality provided by others in that actor’s community.

### Athletic video

In most studies, we used the prosocial video in an experimental condition, contrasting it to a control Athletic video, chosen to be similarly enjoyable and entertaining but to contain far fewer cues of prosociality. Like *Unsung Hero*, this video features high production quality, a focal young man performing exceptional acts in an urban environment, and evocative music. Instead of a series of charitable and helpful actions, the protagonist performs parkour, a type of street acrobatics. Other elevation researchers have similarly contrasted prosocial stimuli with athletic or artistic displays as positively-valenced control conditions [12,49]; we chose parkour because viewers can readily recognize the skills required to, for example, run up a wall and perform a back flip, and thus do not require any understanding of a specific artistic or competitive context to appreciate the extraordinary nature of the performance. (In the General Discussion we consider implications of minor ambiguous cues to prosociality in this video: the impressive physical performance seems intended to entertain viewers, and the fact of the video being made and watched suggests that the performer has achieved some social status or rewards derived from diligent training efforts.)

### Neutral video

In several studies, we implemented methods wherein, after completing the baseline idealism scale, participants watched a neutral video and then completed an elevation scale. This was done in order to: (1) attempt to reduce any variation in participants’ baseline emotion state (due to experiences just prior to participation, etc.); (2) familiarize participants with the elevation scale; and (3) facilitate within-subject comparisons of elevation measures prior to and after the primary experimental manipulation.

The Neutral video features 30 seconds of footage filmed in a commuter train occupied by a few unremarkable passengers who exhibit no overt exceptional behavior. It thus lacks the entertaining and mildly prosocial features of our Athletic video. Thus, should any minor variation in elevation response to the Neutral video be observed, this would more likely be to be due to methods issues (imperfect scale items, inattentive participants, etc.) rather than meaningful individual differences indexed by idealistic attitudes.

### Attitude measure: Idealism-cynicism scale(s)

We conceptualize idealistic attitudes as baseline internal regulatory variables that are consulted when processing potentially elevating social scenarios, variables that are updated based on experience in a somewhat Bayesian process. In light of theorizing that attitudes can be updated by emotional experiences [11], we always measured idealism before participants were exposed to video stimuli; in some studies, we also measured idealism after said exposure.

We attempted to measure idealism with respect to people in general, rather than as an attitude towards specific individuals as in Gervais and Fessler’s model (more on this in the General Discussion). While the details of the idealism measure were refined over the course of the studies reported here, all versions of the scale show good internal reliability (alphas between .82 and .93 in our online studies, and between .73 and .86 in our field studies) and can be straightforwardly interpreted as measuring the extent to which generic others are expected to behave prosocially versus exploitatively toward each other and/or towards the self, e.g., “people [in general] try to be fair,”“I prefer to keep a distance from most people in my broader community”. S1 Appendix includes full details of the various scale versions used here, plus a description of an informal scale development effort based on data from pilot studies (also openly available).

### Emotion measure: Elevation scale(s)

Our elevation scale, largely derived from scales used in prior work in this area [12,13,15,16,50,51], was refined over the course of our initial studies. Despite varying somewhat in their details, all versions of the scale have high internal reliability (full scale alphas > 0.95, subscale alphas > 0.80) and all versions include the same three conceptual subscales as reviewed above: terms describing somatic responses (e.g., “tears in the eyes”), folk terms for related affects (e.g., “moved”), and statements describing prosocial motivations (e.g., “want to help”). See S1 Appendix for all items.

Along with elevation items, we included several items measuring general *positive affect* (e.g., “entertained”, “happy”), an emotion variable which serves as a contrast in several analyses.

Initially following methods of other investigators, our earliest studies presented elevation items as “right now I feel [item],” while subsequent studies framed the items as “the video made me feel [item].” We prefer the latter framing as a way to focus participants’ reports on the emotion induced by the video specifically; our concern with the former framing is that it might suggest that participants should reflect upon the entire experience of participating in the research study (see S1 Appendix). Similarly, our initial efforts included a “view of humanity” subscale which was later removed because our primary focus here is to assess the impact of baseline attitudes on elevation, rather than to explore updates to attitudes resulting from the experience of elevation.

### Behavior measures

Shortly after completing the elevation scale, in many studies participants’ prosocial behavior was gauged using a variety of measures, as follows:

#### Real charitable donation

In field studies, wherein passers-by participated in research in exchange for cash payment, participants had the opportunity to donate any or all of their payment to the UCLA Children’s Hospital via an anonymous donation procedure. Participants recorded their intended donation amount, if any, on the last item in the anonymous survey administered via tablet computer; participants then received their cash payment and, while the researcher turned away, placed any donation into a padded envelope and sealed it. The researcher then marked the outside of the envelope with a code that could be matched to the questionnaire responses; a different researcher later logged the contents of the envelopes, thus ensuring that donation decisions were truly anonymous. Although care was exercised in processing donated funds, the procedure of matching coded envelopes to electronic data left room for human error; thus, the amount pledged by the participant is arguably a more infallible data point than is the amount donated. However, the former is an imperfect approximation of the actual donation, as participants were free to donate any amount regardless of their pledge, and some openly claimed to have donated a different amount from that pledged (a few even gave more than their participation fee, which was not an available pledge option). We analyzed both pledges and recorded actual donations, finding that they are highly correlated and produce similar results. In the main text we report analyses based on actual donations; see S2 Appendix for analyses of pledges.

#### Hypothetical charitable donation

Our online studies used the MTurk employment platform, hence, instead of being compensated volunteers, participants were paid workers. Even the implied suggestion that MTurk workers donate a portion of their wages could be considered inconsistent with the nature of their engagement. Accordingly, rather than asking whether they wished to donate real money to a charitable cause, we instead asked how likely they would be to choose the charitable option in a hypothetical scenario wherein a new employer offered to automatically deduct and distribute charitable donations from employee salaries.

#### Written messages

Because Internet users frequently provide feedback or express opinions without compensation, we felt that it would not violate expectations associated with MTurk employment to present MTurk participants with an optional opportunity to provide free-response feedback, expressing their thoughts about specific topics. Since these responses were not directly compensated, providing them was costly behavior by participants in response to a request for help. These responses were coded to produce quantitative variables, as follows: *feedback provided* (yes/no), *task performance*, and *apparent friendliness*. The latter two variables were subjectively coded by research assistants who were partially blind to experimental condition (the content of some responses revealed the condition) but were not blind to the hypothesis at issue.

*Task performance* was a subjective evaluation of how well the participant answered the question posed, akin to a grade on a short-essay exam. Criteria included apparent effort, thoroughness, and thoughtfulness, but not language skills, rater’s feelings about or intellectual evaluation of the content, or the friendliness of the content. *Friendliness* was a subjective evaluation of whether the tone and content of the message suggested that the participant sought to initiate or preserve a positive relationship with the researchers. Criteria included politeness, compliments/enthusiasm about the study or researchers, and demonstrations of own prosociality, but not task performance.

The specific free response prompts varied between and/or within studies. Four to six research assistants coded every response. We evaluated interrater reliability based on one-way intraclass correlations (ICCs) [52] calculated in R; when topic of the response varied within studies, the data were evaluated separately. ICC values can be interpreted as roughly similar to correlation coefficients. As detailed in Table 2, ICCs for Performance were between .60 and .75, which can be subjectively characterized as moderate or good consistency, whereas Friendliness ratings showed poor consistency. Results based on friendliness scores should thus be interpreted with caution.

**Table 2:** Intraclass correlations for ratings of Performance and Friendliness.

Study (topic)	n raters	Performance ICC [95% CIs]	Friendliness ICC [95% CIs]
**study01**	5	0.63 [0.58 0.68]	0.50 [0.44 0.55]
**study02**	5	0.75 [0.71 0.79]	0.21 [0.15 0.27]
**study03**	4	0.60 [0.55 0.64]	0.00 [-0.04 0.04]
**study11**	5	0.70 [0.64 0.76]	0.29 [0.21 0.37]
**study12**	5	0.70 [0.64 0.76]	0.29 [0.21 0.37]
**study06 (topic 1)**	6	0.66 [0.61 0.70]	0.12 [0.08 0.16]
**study06 (topic 2)**	5	0.61 [0.55 0.66]	0.35 [0.29 0.42]
**study07 (topic 1)**	5	0.70 [0.65 0.74]	0.15 [0.09 0.20]
**study07 (topic 2)**	6	0.70 [0.65 0.75]	0.34 [0.28 0.42]

### Hypotheses and analysis

Because all studies used very similar methods, simply pooling all data for most analyses is a reasonable option. Alternatively, analyses can be conducted separately on data from each study and then the effects meta-analyzed. We conducted analyses based on both approaches in R [53], using the *metafor* package [54], yielding the same qualitative conclusions. In the main text we present visuals based on pooled data (for study-level versions of these figures, see S2 Appendix) and describe meta-analytically derived effect size estimates.

### Elevation as mediator of prosocial contagion

Previous research suggests that elevation mediates the effect of exposure to prosocial scenarios on prosocial behavior. To evaluate whether our studies replicate this finding, we used the R *mediation* package [55] to conduct mediation analyses with elevation (as a function of experimental condition: Prosocial or Athletic) as the mediator, and behaviors (as a function of condition and elevation) as the outcomes. We report the total effects (tau), which contrast behavior in the two conditions such that positive effects show prosocial contagion, and the proportion of these effects mediated by the emotion. We conduct alternate versions of these analyses using each of the elevation subscales rather than the full elevation scale, and contrast those results with models using general positive affect instead of elevation measures.

### Idealism as a moderator of the relationship between condition and elevation

Our novel perspective predicts that differences in elevation response to the same prosocial stimulus should be driven in part by differences in baseline idealism, such that people who expect others to behave more cooperatively will be more elevated. Thus, among people exposed to a video that contains strong candidate cues of prosociality (Prosocial video), there should be a significant correlation between idealism and elevation. For exposure to videos with little (Athletic video) or no (Neutral video) prosocial content, the elevation system should not be activated, such that any variation in self-report measures of elevation should reflect methodological imprecision (e.g., noise) and not the influence of idealism. We can estimate the relationship between baseline idealism and measures of elevation following all three video stimuli, and then compare these effect sizes. While the methods of the studies generating these effect size estimates are not perfectly uniform, we are not aware of any methodological asymmetries that would undermine the validity of such comparison. Alternative analyses can be done at the study level: in studies experimentally contrasting our Prosocial and Athletic conditions, this predicts an interaction between condition and idealism on elevation such that idealism is a stronger predictor of elevation in the Prosocial condition than the Athletic condition.

## Results

Before presenting analyses related to our novel hypothesis concerning the relationship between idealistic attitudes and elevation, we first report results that establish that these methods replicate previous findings about the relationships between exposure to prosocial stimuli, self-reports of elevation, and subsequent prosocial behavior. We also show that elevation is distinguishable from mere positive affect.

### Replication of previous elevation findings

#### Responses to elevation scale and its subscales vary as a function of eliciting stimuli, and are distinguishable from positive affect

**Fig 2:**
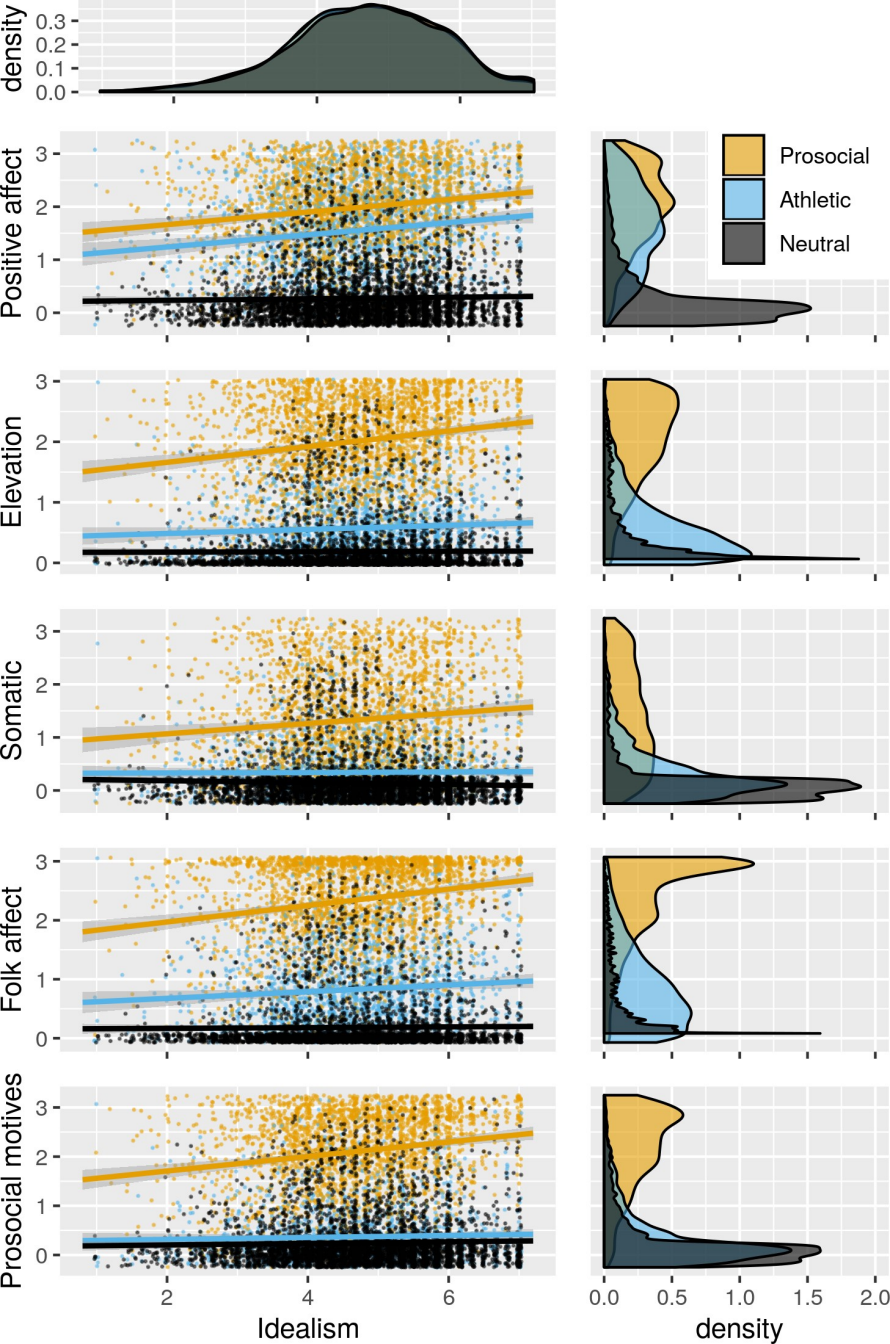
Emotion as a function of idealism and condition. Emotion measures include general positive affect, elevation, and three elevation subscales. Data pooled from 13 relevant studies (total observations: Neutral = 5040, Athletic = 2454, Prosocial = 2619).

Our data set includes *k* = 29 effect estimates of elevation response following exposure to one of our three video stimuli. Among these, type of stimuli is a significant moderator of the effect of video exposure on elevation level (*QM* = 2,020.92, *p* < .001), an effect clearly visible in Fig 2. The Neutral stimulus (*k* = 7) results in near-zero elevation (on 0–3 scale, raw estimate = 0.20 [0.13–0.27]); the Athletic control video (*k* = 11) causes slightly higher elevation (0.57 [0.55–0.59]); and the Prosocial video (*k* = 11) causes much stronger elevation (2.01 [1.93–2.09]). This pattern of elevation differs from that of general positive affect, which also differs by stimuli (*QM* = 990.72, *p* < .001); is also very low in response to the Neutral video 0.28 [0.22–0.35]); and is also high in response to the Prosocial video (1.99 [1.91–2.07]). Positive affect (1.56 [1.48–1.63]) is fairly high following the Athletic video, indicating that this stimulus serves its intended purpose as a positively-valenced control condition.

As intended, there are consistent, dramatic differences wherein elevation scores are markedly higher in the Prosocial condition than in the Athletic condition. That said, elevation scores in the latter are not zero. Examining scores on the subscales of the elevation measure reveals that the (low) levels of elevation reported in the Athletic condition stem largely from the emotion terms. This is understandable given that folk affect labels such as “inspired” and “moved” have broad referents (see General Discussion), underscoring the importance of using multiple methods of reporting subjective experiences. That said, it is worth noting that participants’ scores on the emotion terms subscale are often at ceiling in the Prosocial condition, suggesting that, despite its incomplete specificity, this subscale has high sensitivity.

#### Behavior varies by condition, consistent with prosocial contagion

**Fig 3:**
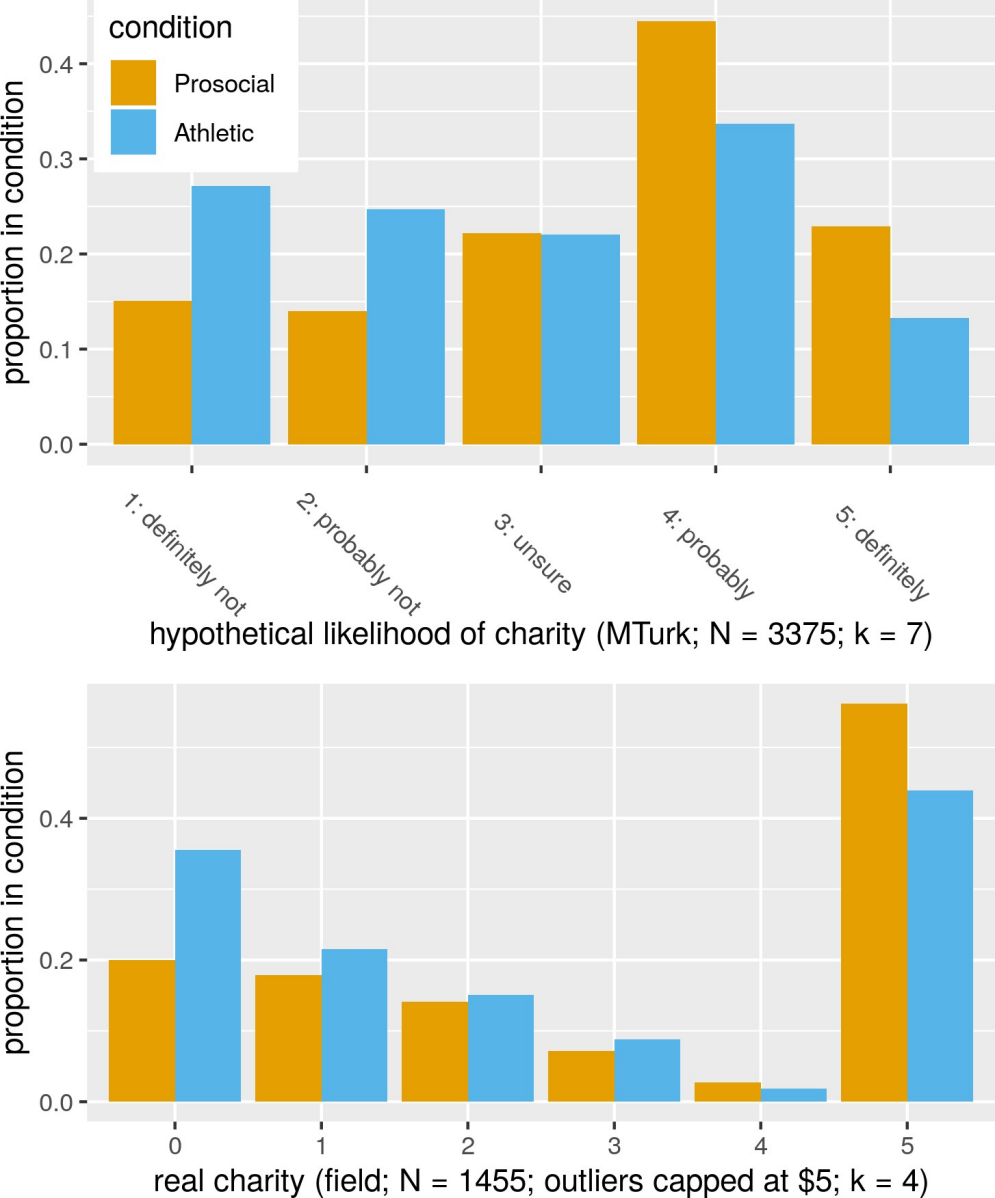
Prosocial contagion: Exposure to a prosocial video is associated with more charitable behavior and intent than exposure to a similarly entertaining control video. Figure pools data from all studies contrasting similar conditions (K = 11).

Our data set includes *k* = 7 studies contrasting the effects of the Athletic and Prosocial videos on the hypothetical charity outcome variable. Visualizing pooled data from these studies reveals a shift towards greater charitability in the Prosocial condition (Fig 3, top panel); the same patterns are present in each study (S2 Appendix). Making the simplifying assumption that numeric Likert scale values can be treated as quantitative, the meta-analytic estimate of the standardized mean difference between conditions on the charity likelihood scale is 0.47 [0.38–0.55]. The corresponding scale mean estimates are 2.78 [2.68–2.88] in the Athletic condition and 3.39 [3.28–3.49] in the Prosocial condition.

For the real charity outcome variable, *k* = 4 studies are available. The shift towards generosity in the Prosocial condition is again visible (Fig 3, bottom panel), and again present in each study and for the charity pledges recorded minutes before the actual charity donation (Supplement); the standardized mean difference in real charitable donation betweens conditions is 0.31 [0.21–0.41]. The corresponding mean donation estimates (in U.S.$) are 2.60 [2.40–2.80] in the Athletic condition and 3.25 [3.10–3.40] in the Prosocial condition.

#### Elevation mediates prosocial contagion

For all of the three behavioral outcomes where prosocial contagion was observed, mediation models suggest that elevation fully mediates the total effect of video condition (Athletic vs. Prosocial) on behavior (Table 3). This is true for analysis of data pooled from all studies and for all study-level charity outcomes (S2 Appendix). The study-level mediation effects of elevation for the friendliness outcomes are all positive, albeit typically with wide confidence intervals (S2 Appendix). Similar results obtain when any of the elevation subscales are used as the mediator variable (S2 Appendix). Of particular importance, not only do self-reported prosocial motives (e.g., “I want to help”) predict actual prosocial behavior (indicating that such self-reports are not merely cheap talk in the service of impression management), but so too do self-reports of both somatic sensations (e.g. “I feel a lump in my throat”) and folk affect terms (e.g., “I feel touched”), suggesting that self-reported motives indeed reflect the motivational facet of an integrated emotion. In marked contrast, positive affect is a far weaker mediator of prosocial contagion.

**Table 3:** Prosocial contagion and mediation thereof.

Behavior	Mediator	n	Tau [95% CI]	Proportion mediated [95% CI]
charity (hypothetical)	elevation	3375	0.60 [0.51–0.68]	1.57 [1.34–1.87]
charity (actual)	elevation	1455	0.64 [0.43–0.85]	0.94 [0.55–1.53]
friendliness	elevation	1681	0.12 [0.05–0.19]	1.29 [0.62–3.01]
charity (hypothetical)	positive affect	3375	0.60 [0.52–0.68]	0.25 [0.20–0.31]
charity (actual)	positive affect	1455	0.64 [0.42–0.86]	0.16 [0.06–0.30]
friendliness	positive affect	1681	0.12 [0.05–0.19]	0.22 [0.09–0.54]

*Note*. Summaries of mediation analyses based on pooling all available data. Displayed are the three behavioral outcomes for which we find positive total effects of the Prosocial Video (compared to Athletic Control, i.e, tau > 0), and the proportion of the total effect that can be attributed to mediation by elevation or positive affect. (See S2 Appendix for similar analyses involving elevation subscales.) When proportion mediated is above 1, this suggests that the mediator fully explains the main effect, with other pathways exerting smaller opposing effects. When this proportion is between 0 and 1, it suggests that the mediator variable partially explains the main effect, with other pathways completing the full effect. Main effects (tau) and confidence intervals for a given behavior are not identical because the mediation analysis involves bootstrapping.

### Idealism explains variation in elevation response, specific to prosocial cues

Having established that our methods reliably induce an emotion state that mediates behavior, we now turn towards our novel question of interest, explaining individual differences in the experience of that emotion.

Fig 2 illustrates that elevation has differing associations with idealistic attitudes across the three types of stimuli. Our data set includes 24 estimates of the correlation coefficient between these variables; meta-analysis reveals stimulus type to be a significant moderator of the effect (*QM* = 50.20, *p*< .001). Elevation is robustly predicted by idealistic attitudes in the Prosocial condition (*k* = 9, 0.22 [0.18–0.26]), whereas the effect is minimal in the Athletic condition (*k* = 9, 0.08 [0.02–0.15]) and absent following the Neutral video (*k* = 6, -0.02 [-0.05–0.02]). The weak positive relationship between idealistic attitudes and elevation scale scores in the Athletic condition is driven largely by the subscale employing folk affect terms (*k* = 9, 0.10 [0.04–0.17]).

In contrast to the idealism-elevation relationship, there was no significant difference in the correlations between idealism and positive affect between the Prosocial (*k* = 8, 0.20 [0.16–0.25]) and Athletic conditions (*k* = 8, 0.15 [0.10–0.21]). In both conditions, idealism predicts positive affect. Speculatively, this may be because appreciating a performance may constitute a form of cooperation, be it with the performer himself, the creator of the video of the performance, or the experimenters who show that video to the participant (see General Discussion).

## General discussion

Building on previous researchers’ work, in a series of 15 studies we experimentally assessed the role of elevation in prosocial contagion. We consistently replicate—and extend—the finding that self-report measures of elevation in response to prosocial stimuli statistically mediate corresponding variation in behavioral responses and motivations; to our knowledge, our field studies are the first to demonstrate this relationship outside of a laboratory or online setting. These findings add convergent validity to a growing body of similar results [8,9].

Further extending these findings, and addressing our key novel hypothesis, results confirm that stronger elevation response is associated with more idealistic attitudes, i.e., greater estimates of the prosociality of other people. Moreover, this attitude-emotion relationship is moderated by context, being evident only in response to our prosocial stimulus–exactly as is to be expected if, rather than simply being a manifestation of an optimistic disposition, idealism is part of a mechanism designed by natural selection to adjust prosocial motivation as a function of the profitability of prosociality in a given situation.

### The utility of the Attitude-Scenario-Emotion model of affective systems

We applied a key principle of Gervais and Fessler’s [11] Attitude-Scenario-Emotion model: the mechanisms governing the deployment of an emotion should be adaptively sensitive to the context-specific costs and benefits of the behaviors motivated by that emotion. In their model, attitudes are the mechanisms that encode various expectations and valuations to allow such emotional calibration. Deductive research on affective systems can therefore distinguish between the emotion and the baseline attitudes that should moderate the deployment of that emotion.

The conceptual distinction between emotion and attitude proved useful for understanding elevation. Previous investigators’ research, which often employs measurement instruments that combine emotional and attitudinal information, can be reinterpreted as indicating that the somatic and motivational symptoms of elevation are associated with feelings and cognitions consistent with idealistic attitudes. Our work clarifies this association. Methodologically, our studies cleave the two constructs, measuring them separately and at different times. Our data show that these variables are associated only in contexts relevant to contagious prosociality. These results are consistent with a mechanistic model whereby idealistic attitudes regulate the deployment of elevation. More work is required to refine and more extensively test the model, and the present methods could be readily adapted to test other implications of our model, or to directly contrast our model with rival models. Future work could investigate the cue processing aspect of our model (Fig 1), which was not directly tested here. For example, idealism could be associated with stronger endorsement of inferences such as “the actor I witnessed was behaving cooperatively,” and “I would benefit from behaving similarly.” It is possible that this functional logic is consciously accessible, although keeping such information out of consciousness might help avoid the negative consequences of appearing Machiavellian [3,6].

### Functional perspectives on elevation

In light of the positive valence of elevation, researchers have often noted elevation’s fit with Fredrickson’s *broaden-and-build theory* [8,56,57], a high-level functional theory, focusing largely on the valence and motivational outputs of emotion systems. This theory holds that, unlike negatively-valenced emotions, positive emotions tend to *broaden* the individual’s set of available behavioral options, which functions to *build* the individual’s personal resources. Gervais and Fessler’s Attitude-Scenario-Emotion model is potentially compatible with broaden-and-build, as the former does not address characteristics of superordinate positive/negative categories of emotions, instead emphasizing form/function fits between the outputs of specific emotion systems and specific adaptive challenges or opportunities implied by the informational inputs to the system. Thus we anchor our model of elevation—an emotion widely agreed to cause cooperative behavior—against functional-level theories of how cooperative behavior can enhance biological fitness, and apply the Attitude-Scenario-Emotion model to understand the adaptive fit between affective mechanisms and the selection pressures implied by the cues that elicit the emotion. Selection favors strategic cooperators over indiscriminate cooperators, so mechanisms that cause cooperative behavior—including elevation–should do so when cues suggest that cooperation is especially likely to be profitable (in the currency of biological inclusive fitness). This interpretation finds common ground with broaden-and-build in that adaptive use of prosociality builds personal resources (or otherwise contributes to fitness-relevant goals), but our theory adds functional specificity regarding the nature of prosociality calibration in response to relevant information, and the process by which this occurs.

Algoe and Haidt [16] suggest that elevation responds to “moral excellence that does not benefit the self” (p 3). (“The self” refers to the witness of the behavior. This distinguishes elevation from gratitude, which is associated with benefits to the self–more on this below.) They argue that elevation motivates the witness to engage in relationship-building with the prestigious virtuous actor, and these relationships facilitate learning by imitation such that the learner eventually gains prestige similarly to the exemplar. While Algoe and Haidt do not explicitly present this verbal model in evolutionary-functional terms, and while our model does not explicitly invoke the concepts of prestige-biased learning or “moral excellence,” the two models are in general agreement on at least three important points, discussed below.

First, despite our largely bypassing the issue of morality, our model is generally consistent with Algoe and Haidt’s framing of the emotion and/or its elictors in moral terms. Indeed, many investigators conceptualize elevation as related to moral judgment. Haidt [7] positions elevation as the opposite of moral disgust; Diessner and colleagues [58] claim that moral beauty elicits elevation. We suggest that the kinds of behavior that elicit elevation can be regarded as on the extreme end of a continuum from mundane to spectacular cooperation; given the centrality of cooperation in most systems of morality [59,60], such actions are likely to be judged as virtuous under a variety of moral schemas. Thus, elevation can be understood as constituting an extreme end of corresponding continua of emotional and cognitive responses to such behavior, along with morally-valenced admiration (more on this below) or, antithetically, condemnation [18]. Beyond this, our framework does not directly engage with moral considerations and theories; instead we focus on a functional analysis of behavioral inputs and outputs that include prosociality. Evolutionary theories of moral judgments [61] often build from the same body of theory about the functions of cooperation on which we base our elevation model, suggesting avenues for further theoretical unification.

Second, we have emphasized that the inputs and outputs of elevation both involve cooperative behavior, a phenomenon we call prosocial contagion. This could also be described as “imitation” of a prosocial strategy. Third, such contagion or imitation is expected to lead to the imitator reaping social benefits similarly to the exemplar.

One notable difference between Algoe and Haidt’s approach and our model is that ours does not require any future relationship between the elevated witness and the exceptionally cooperative actor(s), though our model accommodates such scenarios among various possible avenues of future cooperation. In our model, the information gained by witnessing the actor is sufficient to recalibrate the system to the likely benefits of a cooperative strategy; a strategic response need not include the exemplar as an ongoing social partner in exchange of information or resources. We are open to the possibility that elevation might be associated with a desire to learn more from the exemplar or otherwise invest in that relationship, but are not aware of any systematic investigation of this issue.

Further, we note two difficulties with Algoe and Haidt’s prestige-biased learning account. One, our methods and those of many other investigators establish that elevation can be induced in situations where the virtuous exemplar is distant or fictional (more on fictional elicitors below), and thus not available as an ongoing social partner. Two, elevation is more strongly associated with diffuse prosocial motives (e.g., “I want to be a better person”) and helpful behaviors directed at various third parties, rather than motivation that more narrowly targets cooperatives efforts towards an exemplar. Whether these two patterns reveal a true structure of elevation–one that is inconsistent with the prestige-biased learning account–or are simply artifacts of research methods deserves further investigation.

### Comparing elevation to other constructs

Although it is increasingly possible to differentiate discrete emotional states using brain imaging [62], such methods are costly and cumbersome, limiting their utility for much basic research on emotions. Correspondingly, many investigations of emotion rely at least in part on participants’ reports of their current (or imagined) emotional state in response to a witnessed (or hypothetical) event [63–66]. In asking subjects to identify or describe qualia (e.g., “How does X make you feel?” etc.), investigators either rely on participants to generate lexical labels for feeling-states, or else employ scales using previously generated labels. While valuable, such methods suffer the limitation that emic emotion terms do not directly correspond with the psychological mechanisms, or even the outputs thereof, at issue. Work in psychological anthropology, cultural psychology, and related fields explores how cultural schemas differentially frame, emphasize, or ignore facets of emotions, and how this shapes not only how participants talk about their emotions, but, moreover, both their experience of emotions and the manner in which emotions impact their behavior [10,67,68]. As such, folk affect terms cannot be expected to carve nature at its joints, but instead embody the complex interaction of biologically-evolved psychological mechanisms and culturally-evolved models of persons, experiences, and social relations. This poses methodological problems for the investigator exploring postulated universal features of affective architecture [10,63], importantly including the pitfall that lexical labels and folk categories often blur the lines among emotions and associated cognitions [10,11].

Likely reflecting phylogenetic and/or ontogenetic processes of serial homology, there are family resemblances within classes of emotions. As a consequence, compounding the problem of cultural models noted above, lexical labels often address features shared by related emotions, such that self-report alone will frequently fail to adequately differentiate discrete underlying mechanisms–a particularly acute problem when, as in the case of English speakers addressing elevation, the given language lacks a distinctive term for the emotion at issue. Identifying discrete underlying mechanisms thus requires triangulation using the combination of eliciting conditions, qualia, and behavioral tendencies/motives [10].

With the above considerations in mind, we turn now to the issue of elevation’s place among other emotions, focusing on comparisons within the family of emotions putatively associated with prosocial behavior: gratitude, admiration, *kama muta*, and awe.

### Gratitude

As mentioned above, gratitude and elevation both involve cooperative behavior as input and output, but can be distinguished empirically, with corresponding functional distinctions. Simplifying slightly, gratitude is experienced when benefits are received from another party; prosocial motives are then directed towards the benefactor. This mechanistic structure fits well with the functional logic of direct reciprocity [4,16]. In contrast, elevation does not fit neatly into this direct-reciprocity logic: at the input level, elevation is typically elicited not by being on the receiving end of benefits, but rather by observing someone else being aided in this manner; at the output level, elevation is associated with prosocial motives directed broadly at other people—or even at society at large—rather than at the benefactor whom one has observed as a third party. Nevertheless, while the distinction between gratitude and elevation is clear, our account of elevation does not inherently constrain elevation elicitation to the observation of acts that do not benefit the self. An exceptionally generous act benefiting the self could present a cue that initiates both gratitude and elevation, driven by the implied functional inferences that both prosociality directed at the benefactor and a more diffuse prosociality are likely to be good investments.

### Admiration

Algoe and Haidt classify elevation, gratitude, and admiration as “other-praising emotions,” where admiration is a response to “extraordinary displays of skill, talent, or achievement,” and elevation is similarly defined, but restricted to extraordinary moral virtue (which, as noted above, we translate roughly as extraordinary cooperative behavior). Linking admiration to the functional logic of prestige-biased learning [43], they suggest avenues by which admiration might lead to prosocial behavior. For example, cooperative behavior directed towards those possessing highly valued skills can be profitable if it facilitates learning from the skillful exemplar, such as in an apprenticeship, where low-skilled help buys access to expertise. Praising an exceptionally skilled exemplar, including moral praise, similarly invests one’s own social capital in increasing the prestige of the exemplar [18], which could be considered a prosocial consequence of admiration.

While admiration thus plays a role in pathways leading to prosocial behavior, we caution against concluding that directing material or social rewards towards the exemplar (or any future direct relationship with the exemplar) should always be the expected response to witnessing extraordinary skill. First, some exemplars dispense their wisdom freely through specific channels but are difficult to access otherwise; in such situations admiration should motivate information-seeking focused on the exemplars but not necessarily direct cooperation with them. Second, some skills may be admired but acquiring them judged to be beyond the observer’s ability or interests. In such cases, admiration might affectively mark that individual’s skills as an important feature of the individual, but further investment in the relationship may not be useful. Third, the information present in an extraordinary display of skill can be exploited without any further cooperative or informational relationship with the exemplar. Just as an extraordinarily prosocial exemplar implies that prosocial behavior can be rewarded in the present social environment, any extraordinary performance of other skills can imply that developing such skills is possible and can be rewarded. In such situations, admiration could motivate independent learning or seeking other exemplars.

In sum, we think that admiration is likely to be a part of the elevation experience because it is a response to extraordinary praiseworthy behavior more generally (indeed, it is an item on our scales), but we do not expect all admiration experiences to motivate prosocial behavior.

### Kama muta

Fiske and colleagues [10,48,69–71] have recently inductively explored an emotion that they label *kama muta*, which is isomorphic with elevation in several ways, including folk terminology (e.g., “moved”), somatic symptoms (e.g., “moist eyes”), and many of the eliciting stimuli (including, among others, the same video from which our prosocial stimulus was created). Like our adaptationist account of elevation, these authors also propose that kama muta functions to regulate cooperative behavior. However, kama muta researchers specify communal sharing as the type of cooperation that is both the input cue and the output motivation, whereas our proposal regarding the function of elevation does not privilege a specific mode of cooperation either as input or as output, and instead focuses on the adaptive fit between eliciting cues and the expected biological fitness value of behavioral outputs. Importantly, these investigators have yet to map the functional logic whereby witnessing communal sharing should favor motivation to engage in communal sharing.

We do note that at least one type of plausibly prosocial behavior measured in our studies–providing optional free response answers in online studies–did not show any evidence of prosocial contagion, so we are open to theoretical refinements that specify certain types of cooperative outputs of the system(s) under investigation. Are elevation and kama muta different labels for the same emotion? Is one a variant, or particular manifestation, of the other? The parallel bodies of work on elevation and kama muta raise important questions for future work.

### Awe

“Awe” is commonly used as part of lay descriptions of the elevation experience. Piff et al. [72] present results that they and others interpret as indicating that awe itself causes prosocial behavior [73]. Prior investigators have usefully characterized awe as a response to an exceptional stimulus beyond the observer’s previous experience or frames of reference, resulting in a need to refine existing mental representations to accommodate the new information [74,75]. It is not clear that such an information-based recalibration process would necessarily favor prosocial output, raising the question of the mechanisms responsible for the results that Piff et al. report. Paralleling our argument regarding admiration, we propose that, rather than being a direct driver of prosocial behavior, in those cases where awe is linked to prosociality, this occurs via indirect causal pathways.

If elevation is elicited by exceptional prosociality (as noted above, this is a characterization unique to our model, but consistent with the moral-virtue account offered by prior investigators), this straightforwardly implicates awe as likely to be a component of the experience. In this view, awe functions as a general-purpose learning mechanism that informs other downstream systems given the presence of important new information, and elevation is one such downstream system. In this sense, awe could be said to cause prosociality, albeit indirectly.

Awe experienced in the context of elevation is positively-valenced and a response to specifically prosocial stimuli, but awe can also be experienced as negative and fear-linked, and is often—if not prototypically–experienced in response to non-social stimuli [74,76]. The informational recalibration construal of awe suggests links to social emotions and behavior in some of these situations. If asocial novel stimuli that induce awe also present challenges that are best solved through cooperative means (e.g., working with neighbors to cope with a powerful natural threat, etc.) or present opportunities to deliver perceived benefits to social partners (e.g., sharing an amazing scenic view with a partner), cooperative behavior is likely to follow. Complementing this functional perspective, Nelson-Coffey et al. (2019) note that previous research on awe’s effects has not adequately accounted for other mechanisms, especially a family of social emotions that the authors show are commonly experienced alongside awe, such as gratitude, compassion, and feelings of social connectedness. This provides a framework for reinterpreting Piff et al.’s results. For example, participants taken to view tall trees reported more awe and behaved more helpfully than participants taken to view tall buildings. Rather than interpret this as evidence that awe causes prosociality, we suggest that prosocial emotions related to the enjoyable awe-inducing experience are the cause here. Participants may have felt gratitude to the experimenter, or elevation because the local community is maintaining a spectacular natural public resource.

Piff et al. [72] also suggest that awe influences prosocial behavior via the feeling that one is small. This could be reconciled with our reinterpretation, to the extent that such feelings are an indirect expression of prosocial attitudes and emotions. Mental representations of size have been shown to index traits relevant to social standing, including prestige [77], so feeling small could be associated with high valuation of others. Although feeling “small” can thus be linked to prosocial behavior, it is not clear that, in general, feeling small is a good fit with approach-oriented prosocial motives. For example, shame is similarly associated with the subjective sense of feeling small [63,78], yet, rather than leading individuals to approach others and interact with them prosocially, it instead motivates withdrawal from social interactions–particularly withdrawal from those who elicited the shame [63,78]; likewise, the shame experience is associated with anger and aggression, the antithesis of prosociality [79].

In sum, we think that awe is likely to be a part of the elevation experience because it is a mechanism that responds to noteworthy or exceptional new information, but we do not expect all awe experiences to motivate prosocial behavior.

### Which prosocial cues induce elevation?

The studies reported here rely exclusively on a single elevating video and two control videos, providing analytical precision, but doing so at the expense of generalizability. *Unsung Hero*, the source of our Prosocial video, is crafted to evoke a strong emotional reaction, and thus offers a useful starting point given our research goals. Nonetheless, in light of our reliance on a single stimulus, generalizing from our results requires caution. Testing the elevating effects of various stimuli can help address questions about the boundaries of the phenomenon, a topic we are exploring in other work [80]. In the next sections, we discuss two critical dimensions of stimulus variety.

#### Fictional vs real scenarios

The modern study of elevation was inspired by Thomas Jefferson’s musings about emotional responses to fictional great deeds [16,17]. Continuing this Jeffersonian thinking, we induced elevation using a story about a fictional prosocial hero. The use of fictional stimuli is common in psychological research, including most work on elevation to date, a method based on the assumption that responses to fiction reflect psychology that evolved for responding to real events. Humans have been carefully examining the distinction between emotional responses to fiction and to reality for thousands of years–Hindu sages long ago described rasa, an experience of “tasting” emotion induced by dramatic illusion [68].

Considering this issue in the context of our model of elevation as a response to informative cues about the social environment, we note that there are informational differences between fictional stimuli and real events. Presenting someone with an evocative narrative is in itself a social act to which the recipient may respond separately or in combination with the response to the stimulus itself. This general “outer frame” issue has been previously raised as a concern in regard to the interpretation of experimental economic games as models for cooperative dilemmas more generally [81–83]. As is true of all such research, the experimental control afforded by presenting stimuli precisely determined by the investigator comes at the cost of reduced ecological validity, underscoring the importance of more naturalistic approaches in future research. We note, however, that, per the observations noted above, sharing stories is an ancient social tradition [84], and that being presented with a story about prosocial behavior might imply that there are real prosocial opportunities (e.g., with the story-teller, whose choice of narrative might reveal her likely cooperativeness, or with other audience members, whose attention to the narrative might reveal their likely cooperativeness), such that the logic of contagious prosociality applies equally well to information from the outer frame. Future work may attempt to disambiguate the extent to which elevation and related prosocial behavior are best understood as a response to the story itself (e.g., because, at least to some extent, we automatically respond to fictional social scenarios as if they were real, akin to salivation in response to pictures of food or sexual arousal in response to pornography) and/or to the social act of sharing a story that includes prosocial content.

#### The type(s) of prosociality cues in the eliciting stimuli

We have thus far emphasized the information value of the presence of an extreme cooperator, but, importantly, multiple cues are likely informative. Our Prosocial video also contains cues about how people respond to the exemplar, cues that are also congruent with elevation elicitation, in that beneficiaries of the protagonists’ actions eventually respond positively. Future work can more carefully consider the dynamic integration of multiple–and possibly incongruent–social cues presented over various time courses with respect to outcomes including emotional response, behavior, and attitude updating. For example, in recent work, we explored how elevation experiences differ as a function of the social actions responding to an initial act by an exceptionally kind actor. We found that elevation is diminished when other people respond to the exemplar in antisocial ways compared to neutral-response scenarios; intriguingly, at least within the constraints of the methods that we employed, elevation is not enhanced when others respond prosocially to the exemplar [80].

Our control Athletic video was intended to be neutral with respect to prosociality, but it is possible that minor, indirect cues of prosociality may be detectable. While acrobatic behavior itself is not inherently prosocial, many types of skilled performances are appreciated and rewarded by audiences, such that (quasi)cooperative relationships can emerge from common interest in non-prosocial activities such as sports or arts [18], or, as suggested above, enjoying awe-inducing scenic views. For example, the production of a video of the performance implies that the athlete has been rewarded for doing something that people enjoy. Relatedly, due to the entertaining nature of the video, the experiment itself might be regarded as unexpectedly pleasant–a minor kindness to the participant performed by the researcher; our Athletic condition could thus have induced a modest degree of elevation relative to the neutral condition via the outer frame.

Alternately, modest increases in elevation scores in the Athletic condition relative to baseline may owe to overlap between emotions (or merely between the relevant folk affect terms, e.g., “inspired”) attending, on the one hand, a positive feeling toward a skilled performer such as admiration, as discussed above (indeed “admiration” is an item on this subscale) and, on the other hand, the more cooperation-specific mechanisms of elevation. Consistent with this explanation, it is the folk affect term subscale of our elevation measure that is primarily responsible for the non-floor elevation scores in the Athletic condition, and for idealism’s slight value as a predictor of elevation response to this video. Participants report being “inspired” by the athlete, and feeling “admiration” for him, but they do not get tears in their eyes, nor are they more motivated to behave prosocially. Potentially compatible with a prestige-biased learning account, feelings of admiration and so on are more likely among idealistic participants. If feeling “inspired” is linked to entertaining the possibility that one might learn from a skilled performer, then believing that others are generally prosocial may be one prerequisite for such feelings, as prosocial actors provide access to learners seeking to emulate them.

Regardless of the causes of the minor effects of the Athletic video on elevation, this stimulus nonetheless performs its methodological function as a positively-valenced control condition, since general positive affect is similar between this and the experimental condition.

### What behaviors does elevation govern?

Like much research on prosociality, we used both real, pledged, and hypothetical financial generosity as dependent measures, producing our strongest evidence of behavioral contagion. In real-world systems, prosociality obviously takes many non-financial forms. In consideration of this, we reported evidence based on subjective evaluation of the content of free-response messages written by participants. Although the results are suggestive that behavioral contagion can take the form of a friendly interaction style, interpersonal friendliness is subject to considerable interpretation, hence our findings should be viewed with caution. We encourage experimenters to employ multiple measures of prosocial behavior; such diversity of methods will be especially useful in resolving theoretical questions regarding specific types of output behaviors.

### Study populations

The current research utilized two different methods of convenience sampling, and found qualitatively similar results both among samples composed of people on the street of a large, cosmopolitan American city and in multiple large national samples of MTurk workers. The extent to which our findings would generalize to underrepresented segments of the U.S. population or to other cultures is an open question. Fiske et al [69] argue that the emotion which they label kama muta occurs across cultures and throughout history; if elevation and kama muta are indeed closely related, or even isomorphic (see discussion above), the current findings may generalize quite broadly. In a separate report [85], we present data from elevation studies we conducted in non-Western countries. The type of diversity most relevant to our theoretical model is along the dimension of idealistic attitudes. In the samples reported here, the distribution of this trait is largely in the more idealistic half of the range of the scale (Fig 2, top panel), as should be expected since the outer frame was a cooperative interaction between researcher and participant. If and when highly cynical people are susceptible to prosocial contagion is an interesting and challenging topic for future study. Lastly, our open data set includes various standard demographic variables available for secondary analysis; e.g., we present an exploratory investigation of sex differences in S2 Appendix.

### Measurement

Recent reviews of elevation research have suggested that standardized measurement scales could be useful [8,9]. We sought to create a reasonably short scale to use in both online and field studies. Despite its brevity, our scale arguably offers improvements over many existing measures, as we designed subscales to allow for analytic parsing of the elevation experience, and excluded attitudinal items that might confound results. Further development of a richer elevation scale with additional subscale items, allowing for more detailed assessments of which aspects of elevation are unique to this emotion and which are shared with other affective experiences, could be valuable. Relatedly, as is true of much research on emotions employing self-report scales, the types of prompts sometimes used in studying elevation (e.g., “right now I feel”) suffer the limitation that participants’ responses appear to blend reactions to the stimulus with reactions to the experimenters (or, at the least, with reactions to the experience of being in the experiment), i.e., this prompt blurs what we term the inner frame and the outer frame. Although participants are unlikely to be fully cognizant of the various inputs determining their current emotional state, nonetheless, explicitly directing participants to think about the stimulus (e.g., “the video made me feel”), as we have done in many of our studies, plausibly reduces the contribution of the outer frame to self-reports.

We measured participants’ idealism towards an extremely general set of people (either “my broader community” or “people in general”). In large-scale societies in which individuals frequently interact with others about whom they know little, such generic attitudes may be useful. However, because adaptive calibration of responses is facilitated by attitudinal accuracy, whenever past experience, socially transmitted information, or both afford discrete attitudes focused on particular classes of others, individuals should possess attitudes with various levels of specificity. Future research may explain more variance in emotion by measuring the relevant idealisms with greater precision (e.g., with regard to in-group members versus others, or, even more precisely, towards specific individuals involved in a scenario, etc.). More broadly, ethnocentric attitudes are obvious candidates for study, and may plausibly be associated with greater elevation in response to in-group prosociality than to similar behavior by out-group members; stereotypes are likewise relevant.

### Comparing idealism to other constructs

We have introduced the novel construct of idealistic attitudes, and have shown that this variable explains variation in elevation, as predicted. Idealistic attitudes likely connect to such phenomena as trust, secure attachment, and agreeableness, among many others. Our approach offers a coherent conceptual foundation for the exploration of such connections. For example, an influential framing of trust defines it as “the willingness of a party to be vulnerable to the actions of another party based on the expectation that the other will perform a particular action important to the trustor, irrespective of the ability to monitor or control that other party” [86]. As we have discussed, prosocial actions both have the potential to enhance the individual’s welfare via direct or indirect reciprocity, and, simultaneously, place the individual at risk of exploitation. Hence, trust in others is central to prosociality as an adaptive strategy, and can be viewed as a key component of the cognitive representation of others that we term idealism. Likewise, the ontogenetic process of attachment and its resulting impact on adult social orientation can be understood as the calibration over developmental experience of the propensity to trust others [87]. Lastly, the personality trait agreeableness can be understood as patterned behavior resulting from a high level of trust in others, a correspondingly high level of idealism, and a resulting propensity toward prosociality [88]. While the present project does not explore the multiplex connections between our own and other models and constructs, as we hope the above discussion makes clear, there is great potential for integration among them.

### Future direction: Other emotions regulated by idealism?

The Attitude-Scenario-Emotion model posits that a given attitude can potentiate or inhibit various emotions in relevant scenarios; this entire network of attitude, scenarios, and links to multiple emotions is labeled a *sentiment*. Besides elevation, what other social emotions may be embedded in the sentiment anchored by idealistic attitudes? Are high-idealists more outraged (or disappointed) by exploitative actors? More sympathetic to victims? More grateful towards benefactors? More ashamed of their own mistakes? More delighted by (or proud of) their friends’ successes? Answering such questions will leverage the substantial potential of the Attitude-Scenario-Emotion approach to illuminating individual differences in emotion and social behavior.

### Harnessing elevation to engineer cooperation

A growing literature provides an empirical basis for the conclusion that elevation is a potentially important factor in the spread of prosocial behavior within groups. This corpus offers an invaluable foundation for research into elevation. However, being largely inductively derived, previous pioneering work in this area appears to have conflated two conceptually distinct phenomena. From a functionalist perspective, willingness to engage in prosocial action should hinge on the profitability of such action in the current social context. The results reported here document that prior expectations of others’ prosociality predict the extent to which exposure to a depiction of a highly prosocial actor elicits elevation, which in turn shapes the observer’s own prosocial behavior. Individuals thus differ in their susceptibility to elevation as a pathway of contagious prosociality. In principle, the effects of experience and socially transmitted information on idealism are such that virtuous cycles can occur (or be created) within groups, resulting in communities that are increasingly prosocial and increasingly emotionally rewarding. Understanding more fully the possibility that individuals harbor multiple idealisms, each directed toward different social categories or geographical locales, will thus likely be important in illuminating cultural and regional differences in prevailing levels of prosocial behavior. Looking forward, a fuller portrait of the relationship between experience, expectations, and emotions may prove critical in our comprehension of the factors leading to the origins and stability of communities in which individuals benefit, rather than exploit, one another.

## Supporting information

S1 Appendix Supplementary procedure. (PDF)

S2 Appendix Supplementary results. (PDF)
