# Supplementary material for: Elevation, an emotion for prosocial contagion, is experienced more strongly by those with greater expectations of the cooperativeness of others
Source: PLoS One. 2019 Dec 4;14(12):e0226071. doi: 10.1371/journal.pone.0226071 (PMC6892489; doi:10.1371/journal.pone.0226071)
Supplement: S1 Appendix — (PDF) [file pone.0226071.s001.pdf]

SUPPLEMENTARY PROCEDURE TO: Elevation, an emotion for prosocial contagion, is experienced more strongly by those with greater expectations of the cooperativeness of others

Abstract

See main text.

Keywords: idealism, cynicism, attitude, cooperation, sentiment model

Word count: -

SUPPLEMENTARY PROCEDURE TO: Elevation, an emotion for prosocial contagion, is experienced more strongly by those with greater expectations of the cooperativeness of others

Contents

Abstract 2

SUPPLEMENTARY PROCEDURE TO: Elevation, an emotion for prosocial contagion, is experienced more strongly by those with greater expectations of the cooperativeness of others 3

Methods Details 4

Lines of research and registration record 4

Studies 1 to 4: "Fundamental studies" 5

Studies 5 to 9: "Attitude Framing studies" 6

Studies 9 and 10: "Social context studies" 6

Studies 11 and 12: "Personality studies" 7

Studies 13 to 15: "Prevalence of prosociality studies" 7

Analysis 8

Recruitment 9

Scales 9

Idealism-Cynicism 9

Elevation 13

Free Response Ratings 15

Reliability analysis approach 15

Alternative reliability analysis yields similar results 16

Software Version and Source Information 18

References 23

Methods Details

All registrations, survey instruments, data, code, and other details are available at:

https://osf.io/cm64n/

Lines of research and registration record

In the main text we note that the studies reported there are drawn from several lines of research, including some focused on ancillary hypotheses. Here we provide more information about those additional lines of research. We also discuss minor deviations from registered plans. For all studies, sample sizes were chosed based on being large compared to previous elevation studies, while balancing this against practical considerations like time and expense. The data collection methods of all studies were conducted as described in registrations, except where studies were cut short because unanticipated operational problems were discovered. Registered hypotheses are generally consistent with the theorizing we present in the main text. Some specific data analysis plans were abandoned, especially once we began to focus on meta-analyses rather than analyses at the study level. Registrations were typically not specific about data cleaning strategies, although we always registered the survey instrument itself, which included attention check items and device useage questions used as filters. For online studies, we excluded participants who finished faster than a reasonable time threshold; this threshold was typically determined after examining actual completion times. For field studies, some exclusion criteria were implemented at various points during protocol adminimstration by research assistants in the field (such potential participants are not included in the "starting sample"); field participants were excluded based on apparent inattentiveness or distractedness, technical difficulties with tablet computers, or other deviations from protocol.

The Open Science Framework page for the current paper is: https://osf.io/cm64n/.

Studies 1 to 4: "Fundamental studies". The core methods were primarily developed in a series of eight studies, four of which merit reporting; four pilot studies were preliminary and/or methodologically flawed, and thus not reported here. The main text reporting includes three MTurk studies and one field study from this early phase.

Three exploratory pilot studies were conducted prior to the eight studies mentioned above; these pilots informed development of scales for measuring elevation and idealism. Pilot Studies 1 and 2 randomly assigned MTurk participants to watch montage videos of either prosocial, antisocial, or neutral behavior, then report their feelings and motivations in free response format, then complete pilot versions of scales. Free responses were used to compile a long list of candidate items for scales, which were then deployed in Pilot 3. Pilot 3 was the first pre-registered study in this line of work, and included the hypothesis that an idealistic attitude would be associated with stronger elevation response. These three pilot studies also included items in which participants were asked to express their feelings through specific metaphors; this aspect of the project was separately preregistered and will be described in a separate report. These studies also investigated emotional and behavioral response to antisocial behavior.

The hypotheses preregistered for (Fundamental) Studies 1 and 2 were essentially the same hypotheses investigated and confirmed in the present report (except for a prediction regarding metaphors, which we will discuss in a forthcoming report). Pilot 4 honed methods involving repeated measures of idealism that were used in (Fundamental) Study 3.

(Fundamental) Study 4 was our first successful attempt to deploy these general methods in a field study; a pilot version of this study was abandoned before reaching half of the planned sample size due to methods shortcomings that were improved for Study 4. These studies pursued the same general goals as previous studies, with some additional narrow goals described in their registrations but not relevant to the current report.

The registrations for Pilot 4 and Study 3 included analysis plans involving structural equation models; these plans were abandoned in favor of the simpler analyses reported here.

Registrations, survey instruments, data, preliminary analysis code, field assistant protocols, and other information about these studies (including pilots) are available at:

https://osf.io/qb4n9/

Studies 5 to 9: "Attitude Framing studies". This series was designed to investigate the relative predictive value of the idealism scale when framed egocentrically (e.g., "I am treated well by my broader community") versus allocentrically (e.g., "People in my broader community treat each other well"). As we report elsewhere (Sparks, Fessler & Holbrook, in prep), both versions have similar relationships with elevation. Framing of the attitude scale can therefore be ignored here, and data from these studies pertaining to core questions are included in the present analyses. This series included three MTurk studies, Study 5, Study 6, and Study 7, and one field study, Study 8. The registration for Studies 6 and 8 included specific analyses very similar to those used in the current report.

Registrations, survey instruments, data, preliminary analysis code, field assistant protocols, and other information about these studies are available at:

https://osf.io/xbdve/

Studies 9 and 10: "Social context studies". A series of three field studies investigated whether elevation-related experiences are influenced by (1) the presence/absence of existing social partners (e.g., friends) during the elevating experience and (2) general motivations to deepen and/or broaden one's social network. This line of work is still open, but some data on hand bear on the questions investigated here.

Pre-registrations for the Social Context series of field studies mention both new goals and hypotheses specific to this series, and our continuing interest in the general goals and hypotheses pursued in the Fundamental Series. The second study in this series is not

included in the current report because all participants were with a social partner while they participated. Included in relevant main text analyses are Social Context Study 1 and Study 3, for present purposes labeled as Studies 9 and 10.

Registrations, survey instruments, data, preliminary analysis code, field assistant protocols, and other information about these studies are available at:

https://osf.io/rqeab/

Studies 11 and 12: "Personality studies". These studies were designed for a forthcoming report on the relationships between constructs of general interest here (elevation, idealism, prosocial behavior) and personality constructs. Personality data are not relevant for present purposes, but data from the core methods are included in relevant analyses. Two Mturk studies are available from this series.

The Social Context series initially included the Personality Studies that later split into a separate line of inquiry. Registration for Personality Study 11 only mentions goals specific to that line of work, as does Study 12, except that we also mention an interest in whether a new version of the idealism scale improves upon the previous scale. While the registrations thus do not directly address current goals, the methods are fully suitable to answering the current questions, so these studies are included in the present report.

We registered a field study in the Personality series, but canceled the study after a few hours in the field because implementing the methods proved infeasible.

Registrations, survey instruments, data, preliminary analysis code, and other information about these studies (including the failed pilot) are available at:

https://osf.io/3w9kf/

Studies 13 to 15: "Prevalence of prosociality studies". This series of online studies diversified the eliciting stimuli beyond the standard Prosocial and Athletic videos

used in most of our work, with specific attention paid to varying cues about the prevalence of prosociality in the local environment. For meta-analyses, we include data only from those methods using our standard versions of the Prosocial, Athletic, and Neutral videos. Other results of these studies are reported elsewhere (Fessler, Sparks, Samore, & Holbrook, 2019)*.

Registrations, survey instruments, data, preliminary analysis code, and other information about these studies are available at:

https://osf.io/vcpyg/

Analysis

We used R (Version 3.6.1; R Core Team, 2018) and the R-packages *BayesFactor* (Version 0.9.12.4.2; Morey & Rouder, 2018), *broom* (Version 0.5.2; Robinson & Hayes, 2018), *circlize* (Version 0.4.8; Gu, Gu, Eils, Schlesner, & Brors, 2014), *citr* (Version 0.3.2; Aust, 2018), *coda* (Version 0.19.3; Plummer, Best, Cowles, & Vines, 2006), *dplyr* (Version 0.8.3; Wickham et al., 2019), *forcats* (Version 0.4.0; Wickham, 2018), *ggplot2* (Version 3.2.1; Wickham, 2016), *ggpubr* (Version 0.2.3; Kassambara, 2018), *gridExtra* (Version 2.3; Auguie, 2017), *irr* (Version 0.84.1; Gamer, Lemon, & <puspendra.pusp22@gmail.com>, 2019), *jpeg* (Version 0.1.8; Urbanek, 2014), *kableExtra* (Version 1.1.0; Zhu, 2019), *lpSolve* (Version 5.6.13.3; Berkelaar & others, 2015), *lubridate* (Version 1.7.4; Grolemund & Wickham, 2011), *magrittr* (Version 1.5; Bache & Wickham, 2014), *MASS* (Version 7.3.51.4; Venables & Ripley, 2002), *Matrix* (Version 1.2.17; Bates & Maechler, 2018), *mediation* (Version 4.4.7; Tingley, Yamamoto, Hirose, Keele, & Imai, 2014; Imai et al., 2010a, 2010b; Imai, Keele, Tingley, & Yamamoto, 2011; Imai & Yamamoto, 2013), *metafor* (Version 2.1.0; Viechtbauer, 2010), *mvtnorm* (Version 1.0.11; Genz & Bretz, 2009), *papaja* (Version 0.1.0.9842; Aust & Barth, 2018), *psych* (Version 1.8.12; Revelle, 2018), *purrr* (Version 0.3.2; Henry & Wickham, 2019), *readODS* (Version 1.6.7; Schutten, Chan, Leeper, & contributors, 2018), *readr* (Version 1.3.1; Wickham et al., 2018), *readxl* (Version 1.3.1; Wickham & Bryan, 2019), *rlist* (Version 0.4.6.1;

Ren, 2016), *sandwich* (Version 2.5.1; Zeileis, 2004, 2006), *stringr* (Version 1.4.0; Wickham, 2019), *tibble* (Version 2.1.3; Müller & Wickham, 2019), *tidyr* (Version 1.0.0; Wickham & Henry, 2018), *tidyverse* (Version 1.2.1; Wickham, 2017), and *yarrr* (Version 0.1.5; Phillips, 2017) for our analyses.

The code that produced all analyses in the main text and supplement is openly available.

Recruitment

MTurk studies were advertised with language such as: "10 minute academic survey about feelings and memory. You may (or may not) watch a short video with audio. Please only accept this HIT if you can do it in a distraction-free environment, and please do not complete this HIT on a phone. You may be ineligible if you have completed a similar HIT for us recently–we will automatically detect and prevent this. Thanks!"

Recruitment methods for field studies are included in the openly-available PDF copies of survey instruments.

Scales

In the course of developing our elevation scale, we initially followed prior investigators in measuring the elevation facets discussed in the main text (somatic response, folk affect terms, prosocial motivations) alongside of items assessing views of other people (e.g., "optimistic about humanity," etc.) that we now interpret as measuring attitudes. We do not include any data from early studies where the interspersing of such items could guide participant intuitions about which internal states to consult and report, minimizing opportunity to disassociate them.

Idealism-Cynicism. As discussed in the main text, our Idealism scale evolved somewhat over the course of our research, but all versions are interpretable as indexing expectations about the prosociality of other people. The full scales used in each study are

available as part of the raw data files in the open archives; for the reader's convenience we present them here as well.

Version 1 (Study 1 & 2).

This measure of baseline attitude about humanity was a 10-item scale. Five items are a slightly modified version of the trust scale of Yamagishi and Yamagishi (1994). The other items are from the trust measure employed by Valenzuela, Park, and Kee (2009). All items are in Likert format and presented in randomized order.

> Please rate your level of agreement with the following statements about people in general. [1 – strongly disagree . . . 7 – strongly agree]

1. Most people are basically dishonest (reverse)
2. Most people are basically good-natured and kind
3. If anything, I trust others
4. Most people do not trust others (reverse)
5. Most people are trustworthy.
6. People try to take advantage of you if they get the chance (reverse)
7. People try to be fair
8. You can't be too careful in dealing with people (reverse)
9. People try to be helpful
10. People are just looking out for themselves (reverse)

Version 2 (Studies 3 & 4, 9, 11, 13 & 14).

For Version 2 of this scale, items that we had previously included in our the elevation scale as a subscale termed "views of humanity" were moved into an attitude scale totaling 15 items, each having a reverse-coded version. Participants complete 15-item blocks of attitude items. Each block was drawn from the 30 items below, included either the positive or negative version of each item, and contained roughly equal numbers of positive and negative

items.

Please rate your level of agreement with the following statements about people in general. [1 – strongly disagree ... 7 – strongly agree]

1. Most people are basically honest / Most people are basically dishonest
2. Most people are basically good-natured and kind / Most people are basically malicious and mean
3. Most people trust others / Most people do not trust others
4. Most people are trustworthy / Most people are not trustworthy
5. People don't try to take advantage of you, even if they get the chance / People try to take advantage of you if they get the chance
6. People try to be fair / People don't try to be fair
7. People try to be helpful / People don't try to be helpful
8. People look out for the interest of others / People are just looking out for themselves
9. If in doubt, I trust others / If in doubt, I do not trust others.
10. You don't have to always be careful in dealing with people / You can't be too careful in dealing with people
11. I am optimistic about humanity / I am pessimistic about humanity
12. People can be good to each other /People cannot be good to each other
13. There is lots of good in the world / There is very little good in the world.
14. The actions of most people are often admirable / The actions of most people are rarely admirable
15. Most people care about only themselves / Most people care about more than just themselves.

Version 3: Study 10, 12.

In Versions 3 and 4, a new framing preface was added, and items were modified:

Please think about the kinds of people who you tend to encounter in your daily life, but don't know very well. These people are not family or friends or someone you've had many conversations with. They are acquaintances or strangers who seem typical of the social circle(s) that you are a part of. We will call these people your "broader community" – please think about this group when you answer these questions.

Please rate your level of agreement with the following statements about your broader community. [1-strongly disagree … 7-strongly agree]

1. Life is beautiful in my broader community.
2. In my broader community, it is important to make sure you are not exploited. *(Reverse)*
3. If there's something I need that my family and close friends cannot help with, I know that my broader community will help.
4. I prefer to keep a distance from most people in my broader community. *(Reverse)*
5. I am treated right by my broader community.
6. My broader community is full of great people.
7. In my broader community, people do not trust each other. *(Reverse)*
8. In my broader community, people do not try to take advantage of you, even if they get the chance.
9. Most people are basically honest in my broader community.
10. I am pessimistic about my broader community. *(Reverse)*

Version 4: Studies 5 to 8, & 15).

Version 4 honed the scale down to a smaller number of items.

Please rate your level of agreement with the following statements about your broader community. [1-strongly disagree … 7-strongly agree]

1. In my broader community, most people are basically dishonest with [each other / me]. *(Reverse)*
2. In my broader community, people are considerate of [the interests of others / my interests].
3. In my broader community, people are good to [each other / me].
4. [I / People in my broader community] prefer to keep [my / their] distance from [people in my broader community / each other]. *(Reverse)*
5. People in my broader community treat [me / each other] right.
6. [I / people] feel accepted and included in my broader community.

Elevation. In our initial studies we presented elevation scale items with the phrase "right now, I feel [item]". However, as noted in the main text, participation in experiments such as these entails two social frames of reference, an inner frame consisting of the stimulus and one's reactions to it, and an outer frame consisting of the ongoing interaction between participant and researcher. Accordingly, in order to direct participants' attention and self-report exclusively to the inner frame within which our experimental manipulations took place, following our initial studies, in the majority of our investigations we prefaced scale items with the phrase "the video made me feel [item]".

All versions of our Elevation scale include the three subscales discussed in the main text: folk affect terms for the emotional experience, somatic experiences associated with the experience, and prosocial motivations resulting from the experience. The earliest version of the scale included a fourth subscale, indexing positive views of humanity. We later judged this subscale to be more appropriately treated as indexing attitude and removed these items from subsequent versions of the emotion scale.

Version 1 (Studies 1 & 2).

In Version 1, many of our scale items are based on the variety of measures in the existing elevation literature; some are novel. Eighteen Likert items are presented as "right

now, I feel _____," and participants rate their feeling on the following scale: 0 – not at all, 1 – slightly, 2 – moderately, 3 – strongly.

Our elevation scale is meant to capture the four facets of elevation described above, plus measures of general positive affect. Views of Humanity items are omited from the calculation of elevation scores in all data reported here.

The items are as follows.

Positive affect [two items]: happy, entertained

Folk Affect Terms [six items]: uplifted, moved, touched, inspired, compassion, admiration

Prosocial motives [four items]: want to help, feel close to other people, want to be a better person, want to find new ways to help

Physical [four items]: warmth in chest, lump in throat, tears in eyes, goosebumps/chills/tingles

Views of humanity [two items]: optimistic about humanity, people can be good to each other

We further assess views of humanity with 3 additional items:

Right now, I feel that: 1. There is lots of good in the world 2. There is some good in the world 3. There is little good in the world 4. There is no good in the world

Right now, I feel that: 1. The actions of most people are admirable 2. The actions of many people are admirable 3. The actions of some people are admirable 4. The actions of few people are admirable

Right now, I feel that: 1. Most people care about more than just themselves 2. Many

people care about more than just themselves 3. Some people care about more than just themselves 4. Few people care about more the just themselves

Version 2 (Studies 3 - 15, with very minor adjustments).

How much did the video make you feel… [0 – not at all, 1 – slightly, 2 – moderately, 3 – strongly]

Positive affect [four items]: happy, entertained, amused, delighted

Folk Affect Terms [seven items]: uplifted, moved, touched, inspired, compassion, admiration, heartwarming feeling (in the metaphorical sense)

Prosocial motives [four items]: want to help, feel close to other people, want to be a better person, want to find new ways to help

Somatic symptoms [four items]: the physical sensation of warmth in chest, lump in throat, tears in eyes, goosebumps/chills/tingles

Free Response Ratings

The instruction and training materials for coders are available at:

https://osf.io/8r7fs/

Reliability analysis approach. Since ratings are ordinal, we calculated kappas (Cohen, 1960), with disagreements weighted by the squared distance from agreement. This method of assessing inter-rater reliability is designed for pairs of raters, but these free-response data were evaluated by four to six raters. Thus, we calculate kappas for all possible pairwise combinations and report the minimum, maximum, and mean of these.

There were within-subject differences in the questions asked of participants in the Attitude Framing Studies, so reliability analyses of those data are split by topic (University or Cars).

Alternative reliability analysis yields similar results. Complementing the ICC analysis of rater reliability presented in the main text, here we present results from a similar analysis based on pairwise kappas. See see Table S1 for summary of these results.

Performance ratings are quite reliable. Among pairwise combinations of raters, the worst show some amount of agreement (kappas > .35), and average pairwise kappas are in the .60 to .75 range. In other words, the raters who most disagreed still trended towards agreement, and most rater pairs tend towards high levels of agreement.

Friendliness ratings are less reliable. The worst pairwise kappas were often near zero, meaning the raters who disagreed most strongly showed very little agreement at all. The highest pairwise kappas are in the .5 to .75 range, meaning the raters who most strongly agreed showed a reasonably strong level of agreement. The average of all pairwise kappas is mostly in the .25 to .35 range. Hence, friendliness is clearly a less reliable score than performance, but still has some agreement, such that the average friendliness ratings deserve to be further analyzed, albeit with caution.

Table 1

(S1) Rater reliability

Study	Rating	Min	Max	Mean
study01	Performance	0.47	0.75	0.63
study02	Performance	0.72	0.82	0.75
study03	Performance	0.49	0.78	0.61
study11	Performance	0.63	0.81	0.72
study12	Performance	0.63	0.81	0.72
study06_cars	Performance	0.51	0.86	0.66
study06_uni	Performance	0.42	0.83	0.65
study07_cars	Performance	0.62	0.82	0.71
study07_uni	Performance	0.35	0.90	0.71
study01	Friendliness	0.40	0.74	0.52
study02	Friendliness	-0.06	0.77	0.27
study03	Friendliness	0.04	0.61	0.21
study11	Friendliness	0.08	0.67	0.31
study12	Friendliness	0.08	0.67	0.31
study06_cars	Friendliness	0.02	0.60	0.25
study06_uni	Friendliness	0.20	0.48	0.37
study07_cars	Friendliness	-0.06	0.55	0.21
study07_uni	Friendliness	0.04	0.66	0.36

Note. Each free response was coded by 4 to 6 raters. Here we report minimum, maximum, and mean values for all available pairwise kappas, for each study. Studies 6 and 7 included two different free response topics, so these are analyzed separately.

Software Version and Source Information

The reproducibility of this document and the main manuscript depends upon a set of R packages. It is possible that future updates to these packages could disrupt the functionality of the code. In anticipation of this possibility, we list the versions and sources of all required packages.

```
## - Session info ----------------------------------------------------------
##  setting  value
##  version  R version 3.6.1 (2019-07-05)
##  os       Ubuntu 19.04
##  system   x86_64, linux-gnu
##  ui       X11
##  language en_US
##  collate  en_US.UTF-8
##  ctype    en_US.UTF-8
##  tz       America/Los_Angeles
##  date     2019-09-26
##
## - Packages --------------------------------------------------------------
##  package      * version    date       lib source
##  acepack        1.4.1      2016-10-29 [1] CRAN (R 3.6.0)
##  assertthat     0.2.1      2019-03-21 [1] CRAN (R 3.6.0)
##  backports      1.1.4      2019-04-10 [1] CRAN (R 3.6.0)
##  base64enc      0.1-3      2015-07-28 [1] CRAN (R 3.6.0)
##  bookdown       0.13       2019-08-21 [1] CRAN (R 3.6.1)
##  boot           1.3-23     2019-07-05 [4] CRAN (R 3.6.1)
##  broom        * 0.5.2      2019-04-07 [1] CRAN (R 3.6.0)
```

```
## callr           3.3.2     2019-09-22 [1] CRAN (R 3.6.1)
## cellranger      1.1.0     2016-07-27 [1] CRAN (R 3.6.0)
## checkmate       1.9.4     2019-07-04 [1] CRAN (R 3.6.1)
## citr          * 0.3.2     2019-08-19 [1] CRAN (R 3.6.1)
## cli             1.1.0     2019-03-19 [1] CRAN (R 3.6.0)
## cluster         2.1.0     2019-06-19 [4] CRAN (R 3.6.1)
## colorspace      1.4-1     2019-03-18 [1] CRAN (R 3.6.0)
## crayon          1.3.4     2017-09-16 [1] CRAN (R 3.6.0)
## data.table      1.12.2    2019-04-07 [1] CRAN (R 3.6.0)
## desc            1.2.0     2018-05-01 [1] CRAN (R 3.6.0)
## devtools        2.2.1     2019-09-24 [1] CRAN (R 3.6.1)
## digest          0.6.21    2019-09-20 [1] CRAN (R 3.6.1)
## dplyr         * 0.8.3     2019-07-04 [1] CRAN (R 3.6.1)
## ellipsis        0.3.0     2019-09-20 [1] CRAN (R 3.6.1)
## evaluate        0.14      2019-05-28 [1] CRAN (R 3.6.1)
## forcats       * 0.4.0     2019-02-17 [1] CRAN (R 3.6.0)
## foreign         0.8-72    2019-08-02 [4] CRAN (R 3.6.1)
## Formula         1.2-3     2018-05-03 [1] CRAN (R 3.6.0)
## fs              1.3.1     2019-05-06 [1] CRAN (R 3.6.0)
## generics        0.0.2     2018-11-29 [1] CRAN (R 3.6.0)
## ggplot2       * 3.2.1     2019-08-10 [1] CRAN (R 3.6.1)
## ggpubr        * 0.2.3     2019-09-03 [1] CRAN (R 3.6.1)
## ggsignif        0.6.0     2019-08-08 [1] CRAN (R 3.6.1)
## glue            1.3.1     2019-03-12 [1] CRAN (R 3.6.0)
## gridExtra     * 2.3       2017-09-09 [1] CRAN (R 3.6.0)
## gtable          0.3.0     2019-03-25 [1] CRAN (R 3.6.0)
## haven           2.1.1     2019-07-04 [1] CRAN (R 3.6.1)
```

```
## Hmisc          4.2-0      2019-01-26 [1] CRAN (R 3.6.0)
## hms            0.5.1      2019-08-23 [1] CRAN (R 3.6.1)
## htmlTable      1.13.2     2019-09-22 [1] CRAN (R 3.6.1)
## htmltools      0.3.6      2017-04-28 [1] CRAN (R 3.6.0)
## htmlwidgets    1.3        2018-09-30 [1] CRAN (R 3.6.0)
## httpuv         1.5.2      2019-09-11 [1] CRAN (R 3.6.1)
## httr           1.4.1      2019-08-05 [1] CRAN (R 3.6.1)
## jsonlite       1.6        2018-12-07 [1] CRAN (R 3.6.0)
## kableExtra   * 1.1.0      2019-03-16 [1] CRAN (R 3.6.0)
## knitr          1.25       2019-09-18 [1] CRAN (R 3.6.1)
## later          0.8.0      2019-02-11 [1] CRAN (R 3.6.0)
## lattice        0.20-38    2018-11-04 [4] CRAN (R 3.6.0)
## latticeExtra   0.6-28     2016-02-09 [1] CRAN (R 3.6.0)
## lazyeval       0.2.2      2019-03-15 [1] CRAN (R 3.6.0)
## lifecycle      0.1.0      2019-08-01 [1] CRAN (R 3.6.1)
## lme4           1.1-21     2019-03-05 [1] CRAN (R 3.6.0)
## lpSolve        5.6.13.3   2019-08-19 [1] CRAN (R 3.6.1)
## lubridate    * 1.7.4      2018-04-11 [1] CRAN (R 3.6.0)
## magrittr     * 1.5        2014-11-22 [1] CRAN (R 3.6.0)
## MASS         * 7.3-51.4   2019-04-26 [4] CRAN (R 3.6.1)
## Matrix       * 1.2-17     2019-03-22 [4] CRAN (R 3.6.1)
## mediation    * 4.4.7      2019-03-16 [1] CRAN (R 3.6.0)
## memoise        1.1.0      2017-04-21 [1] CRAN (R 3.6.0)
## metafor      * 2.1-0      2019-05-14 [1] CRAN (R 3.6.0)
## mime           0.7        2019-06-11 [1] CRAN (R 3.6.1)
## miniUI         0.1.1.1    2018-05-18 [1] CRAN (R 3.6.0)
## minqa          1.2.4      2014-10-09 [1] CRAN (R 3.6.0)
```

```
## mnormt          1.5-5      2016-10-15 [1] CRAN (R 3.6.0)
## modelr          0.1.5      2019-08-08 [1] CRAN (R 3.6.1)
## munsell         0.5.0      2018-06-12 [1] CRAN (R 3.6.0)
## mvtnorm       * 1.0-11     2019-06-19 [1] CRAN (R 3.6.1)
## nlme            3.1-141    2019-08-01 [4] CRAN (R 3.6.1)
## nloptr          1.2.1      2018-10-03 [1] CRAN (R 3.6.0)
## nnet            7.3-12     2016-02-02 [4] CRAN (R 3.6.0)
## papaja        * 0.1.0.9842 2019-05-10 [1] Github (crsh/papaja@b6cd70f)
## pillar          1.4.2      2019-06-29 [1] CRAN (R 3.6.1)
## pkgbuild        1.0.5      2019-08-26 [1] CRAN (R 3.6.1)
## pkgconfig       2.0.3      2019-09-22 [1] CRAN (R 3.6.1)
## pkgload         1.0.2      2018-10-29 [1] CRAN (R 3.6.0)
## prettyunits     1.0.2      2015-07-13 [1] CRAN (R 3.6.0)
## processx        3.4.1      2019-07-18 [1] CRAN (R 3.6.1)
## promises        1.0.1      2018-04-13 [1] CRAN (R 3.6.0)
## ps              1.3.0      2018-12-21 [1] CRAN (R 3.6.0)
## psych         * 1.8.12     2019-01-12 [1] CRAN (R 3.6.0)
## purrr         * 0.3.2      2019-03-15 [1] CRAN (R 3.6.0)
## R6              2.4.0      2019-02-14 [1] CRAN (R 3.6.0)
## RColorBrewer    1.1-2      2014-12-07 [1] CRAN (R 3.6.0)
## Rcpp            1.0.2      2019-07-25 [1] CRAN (R 3.6.1)
## readODS       * 1.6.7      2018-11-26 [1] CRAN (R 3.6.0)
## readr         * 1.3.1      2018-12-21 [1] CRAN (R 3.6.0)
## readxl        * 1.3.1      2019-03-13 [1] CRAN (R 3.6.0)
## remotes         2.1.0      2019-06-24 [1] CRAN (R 3.6.1)
## rlang           0.4.0      2019-06-25 [1] CRAN (R 3.6.1)
## rlist         * 0.4.6.1    2016-04-04 [1] CRAN (R 3.6.0)
```

```
## rmarkdown      1.15      2019-08-21 [1] CRAN (R 3.6.1)
## rpart          4.1-15    2019-04-12 [4] CRAN (R 3.6.1)
## rprojroot      1.3-2     2018-01-03 [1] CRAN (R 3.6.0)
## rstudioapi     0.10      2019-03-19 [1] CRAN (R 3.6.0)
## rvest          0.3.4     2019-05-15 [1] CRAN (R 3.6.0)
## sandwich     * 2.5-1     2019-04-06 [1] CRAN (R 3.6.0)
## scales         1.0.0     2018-08-09 [1] CRAN (R 3.6.0)
## sessioninfo    1.1.1     2018-11-05 [1] CRAN (R 3.6.0)
## shiny          1.3.2     2019-04-22 [1] CRAN (R 3.6.0)
## stringi        1.4.3     2019-03-12 [1] CRAN (R 3.6.0)
## stringr      * 1.4.0     2019-02-10 [1] CRAN (R 3.6.0)
## survival       2.44-1.1  2019-04-01 [4] CRAN (R 3.6.1)
## testthat       2.2.1     2019-07-25 [1] CRAN (R 3.6.1)
## tibble       * 2.1.3     2019-06-06 [1] CRAN (R 3.6.1)
## tidyr        * 1.0.0     2019-09-11 [1] CRAN (R 3.6.1)
## tidyselect     0.2.5     2018-10-11 [1] CRAN (R 3.6.0)
## tidyverse    * 1.2.1     2017-11-14 [1] CRAN (R 3.6.0)
## usethis        1.5.1     2019-07-04 [1] CRAN (R 3.6.1)
## vctrs          0.2.0     2019-07-05 [1] CRAN (R 3.6.1)
## viridisLite    0.3.0     2018-02-01 [1] CRAN (R 3.6.0)
## webshot        0.5.1     2018-09-28 [1] CRAN (R 3.6.0)
## withr          2.1.2     2018-03-15 [1] CRAN (R 3.6.0)
## xfun           0.9       2019-08-21 [1] CRAN (R 3.6.1)
## xml2           1.2.2     2019-08-09 [1] CRAN (R 3.6.1)
## xtable         1.8-4     2019-04-21 [1] CRAN (R 3.6.1)
## yaml           2.2.0     2018-07-25 [1] CRAN (R 3.6.0)
## zeallot        0.1.0     2018-01-28 [1] CRAN (R 3.6.0)
```

```
##  zoo            1.8-6      2019-05-28 [1] CRAN (R 3.6.1)
##
## [1] /home/adam/R/x86_64-pc-linux-gnu-library/3.6
## [2] /usr/local/lib/R/site-library
## [3] /usr/lib/R/site-library
## [4] /usr/lib/R/library
```

References

Auguie, B. (2017). *GridExtra: Miscellaneous functions for "grid" graphics.* Retrieved from https://CRAN.R-project.org/package=gridExtra

Aust, F. (2018). *Citr: 'RStudio' add-in to insert markdown citations.* Retrieved from https://CRAN.R-project.org/package=citr

Aust, F., & Barth, M. (2018). *papaja: Create APA manuscripts with R Markdown.* Retrieved from https://github.com/crsh/papaja

Bache, S. M., & Wickham, H. (2014). *Magrittr: A forward-pipe operator for r.* Retrieved from https://CRAN.R-project.org/package=magrittr

Bates, D., & Maechler, M. (2018). *Matrix: Sparse and dense matrix classes and methods.* Retrieved from https://CRAN.R-project.org/package=Matrix

Berkelaar, M., & others. (2015). Retrieved from https://CRAN.R-project.org/package=lpSolve

Cohen, J. (1960). A coefficient of agreement for nominal scales. *Educational and Psychological Measurement*, *20*(1), 37–46. doi:10.1177/001316446002000104

Fessler, D. M. T., Sparks, A. M., Samore, T., & Holbrook, C. (2019). Does observing reciprocity or exploitation affect elevation, a mechanism driving prosociality?

Evolutionary Human Sciences, *1*. doi:10.1017/ehs.2019.3

Gamer, M., Lemon, J., & <puspendra.pusp22@gmail.com>, I. F. P. S. (2019). *Irr: Various coefficients of interrater reliability and agreement*. Retrieved from https://CRAN.R-project.org/package=irr

Genz, A., & Bretz, F. (2009). *Computation of multivariate normal and t probabilities*. Heidelberg: Springer-Verlag.

Grolemund, G., & Wickham, H. (2011). Dates and times made easy with lubridate. *Journal of Statistical Software*, *40*(3), 1–25. Retrieved from http://www.jstatsoft.org/v40/i03/

Gu, Z., Gu, L., Eils, R., Schlesner, M., & Brors, B. (2014). Circlize implements and enhances circular visualization in r. *Bioinformatics*, *30*(19), 2811–2812.

Henry, L., & Wickham, H. (2019). *Purrr: Functional programming tools*. Retrieved from https://CRAN.R-project.org/package=purrr

Imai, K., Keele, L., & Tingley, D. (2010a). A general approach to causal mediation analysis. *Psychological Methods*, *15*(4), 309–334. Retrieved from http://imai.princeton.edu/research/BaronKenny.html

Imai, K., Keele, L., Tingley, D., & Yamamoto, T. (2011). Unpacking the black box of causality: Learning about causal mechanisms from experimental and observational studies. *American Political Science Review*, *105*(4), 765–789. Retrieved from http://imai.princeton.edu/research/mediationP.html

Imai, K., Keele, L., & Yamamoto, T. (2010b). Identification, inference, and sensitivity analysis for causal mediation effects. *Statistical Science*, *25*(1), 51–71. Retrieved from http://imai.princeton.edu/research/mediation.html

Imai, K., & Yamamoto, T. (2013). Identification and sensitivity analysis for multiple causal

mechanisms: Revisiting evidence from framing experiments. *Political Analysis*, *21*(2), 141–171. Retrieved from http://imai.princeton.edu/research/medsens.html

Kassambara, A. (2018). *Ggpubr: 'Ggplot2' based publication ready plots.* Retrieved from https://CRAN.R-project.org/package=ggpubr

Morey, R. D., & Rouder, J. N. (2018). *BayesFactor: Computation of bayes factors for common designs.* Retrieved from https://CRAN.R-project.org/package=BayesFactor

Müller, K., & Wickham, H. (2019). *Tibble: Simple data frames.* Retrieved from https://CRAN.R-project.org/package=tibble

Phillips, N. (2017). *Yarrr: A companion to the e-book "yarrr!: The pirate's guide to r".* Retrieved from https://CRAN.R-project.org/package=yarrr

Plummer, M., Best, N., Cowles, K., & Vines, K. (2006). CODA: Convergence diagnosis and output analysis for mcmc. *R News*, *6*(1), 7–11. Retrieved from https://journal.r-project.org/archive/

R Core Team. (2018). *R: A language and environment for statistical computing.* Vienna, Austria: R Foundation for Statistical Computing. Retrieved from https://www.R-project.org/

Ren, K. (2016). *Rlist: A toolbox for non-tabular data manipulation.* Retrieved from https://CRAN.R-project.org/package=rlist

Revelle, W. (2018). *Psych: Procedures for psychological, psychometric, and personality research.* Evanston, Illinois: Northwestern University. Retrieved from https://CRAN.R-project.org/package=psych

Robinson, D., & Hayes, A. (2018). *Broom: Convert statistical analysis objects into tidy tibbles.* Retrieved from https://CRAN.R-project.org/package=broom

Schutten, G.-J., Chan, C.-h., Leeper, T. J., & contributors. (2018). *ReadODS: Read and write ods files*. Retrieved from https://CRAN.R-project.org/package=readODS

Tingley, D., Yamamoto, T., Hirose, K., Keele, L., & Imai, K. (2014). mediation: R package for causal mediation analysis. *Journal of Statistical Software*, *59*(5), 1–38. Retrieved from http://www.jstatsoft.org/v59/i05/

Urbanek, S. (2014). *Jpeg: Read and write jpeg images*. Retrieved from https://CRAN.R-project.org/package=jpeg

Valenzuela, S., Park, N., & Kee, K. F. (2009). Is there social capital in a social network site?: Facebook use and college students' life satisfaction, trust, and participation. *Journal of Computer-Mediated Communication*, *14*(4), 875–901. doi:10.1111/j.1083-6101.2009.01474.x

Venables, W. N., & Ripley, B. D. (2002). *Modern applied statistics with s* (Fourth.). New York: Springer. Retrieved from http://www.stats.ox.ac.uk/pub/MASS4

Viechtbauer, W. (2010). Conducting meta-analyses in R with the metafor package. *Journal of Statistical Software*, *36*(3), 1–48. Retrieved from http://www.jstatsoft.org/v36/i03/

Wickham, H. (2016). *Ggplot2: Elegant graphics for data analysis*. Springer-Verlag New York. Retrieved from http://ggplot2.org

Wickham, H. (2017). *Tidyverse: Easily install and load the 'tidyverse'*. Retrieved from https://CRAN.R-project.org/package=tidyverse

Wickham, H. (2018). *Forcats: Tools for working with categorical variables (factors)*. Retrieved from https://CRAN.R-project.org/package=forcats

Wickham, H. (2019). *Stringr: Simple, consistent wrappers for common string operations*. Retrieved from https://CRAN.R-project.org/package=stringr

Wickham, H., & Bryan, J. (2019). *Readxl: Read excel files.* Retrieved from https://CRAN.R-project.org/package=readxl

Wickham, H., François, R., Henry, L., & Müller, K. (2019). *Dplyr: A grammar of data manipulation.* Retrieved from https://CRAN.R-project.org/package=dplyr

Wickham, H., & Henry, L. (2018). *Tidyr: Easily tidy data with 'spread()' and 'gather()' functions.* Retrieved from https://CRAN.R-project.org/package=tidyr

Wickham, H., Hester, J., & Francois, R. (2018). *Readr: Read rectangular text data.* Retrieved from https://CRAN.R-project.org/package=readr

Yamagishi, T., & Yamagishi, M. (1994). Trust and commitment in the United States and Japan. *Motivation and Emotion*, *18*(2), 129–166. doi:10.1007/BF02249397

Zeileis, A. (2004). Econometric computing with HC and HAC covariance matrix estimators. *Journal of Statistical Software*, *11*(10), 1–17. doi:10.18637/jss.v011.i10

Zeileis, A. (2006). Object-oriented computation of sandwich estimators. *Journal of Statistical Software*, *16*(9), 1–16. doi:10.18637/jss.v016.i09

Zhu, H. (2019). *KableExtra: Construct complex table with 'kable' and pipe syntax.* Retrieved from https://CRAN.R-project.org/package=kableExtra
