# Supplementary material for: Elevation, an emotion for prosocial contagion, is experienced more strongly by those with greater expectations of the cooperativeness of others
Source: PLoS One. 2019 Dec 4;14(12):e0226071. doi: 10.1371/journal.pone.0226071 (PMC6892489; doi:10.1371/journal.pone.0226071)
Supplement: S2 Appendix — (PDF) [file pone.0226071.s002.pdf]

SUPPLEMENTARY RESULTS TO: Elevation, an emotion for prosocial contagion, is experienced more strongly by those with greater expectations of the cooperativeness of others

Abstract

See main text.

Keywords: idealism, cynicism, attitude, cooperation, sentiment model

Word count: -

SUPPLEMENTARY RESULTS TO: Elevation, an emotion for prosocial contagion, is experienced more strongly by those with greater expectations of the cooperativeness of others

Contents

Abstract **2**

SUPPLEMENTARY RESULTS TO: Elevation, an emotion for prosocial contagion, is experienced more strongly by those with greater expectations of the cooperativeness of others **3**

Results Guide **3**

Summary tables of mediation models . . . 3
Study level versions of main text behavior summary figure . . . 14
Pledged versus actual charitable donation . . . 14
Study level versions of main text emotion and attitude summary plots . . . 14
Contrasting the attitudes and emotions of the two study populations . . . 14
Sex differences in attitude and emotion . . . 14
Framing of emotion scale items . . . 14
Scale reliabilities . . . 16

Figures **18**

Results Guide

Summary tables of mediation models

Tables S1 to S5 summarize results of various mediation analyses in greater detail than the main text summary.

Table 1

(S2) Prosocial contagion and mediation thereof by elevation.

study	behavior	mediator	n	tau [95% CI]	proportion mediated [95 % CI]
study02	charity (hypothetical)	elevation	494	0.57 [0.34 - 0.80]	1.25 [0.84 - 2.12]
study03	charity (hypothetical)	elevation	569	0.39 [0.19 - 0.60]	2.56 [1.55 - 5.18]
study05	charity (hypothetical)	elevation	456	0.77 [0.55 - 1.00]	1.18 [0.79 - 1.76]
study06	charity (hypothetical)	elevation	689	0.64 [0.45 - 0.82]	1.37 [0.94 - 2.02]
study07	charity (hypothetical)	elevation	727	0.59 [0.41 - 0.79]	1.86 [1.37 - 2.70]
study11	charity (hypothetical)	elevation	184	0.80 [0.41 - 1.17]	1.94 [1.08 - 3.82]
study12	charity (hypothetical)	elevation	256	0.51 [0.19 - 0.85]	1.97 [1.04 - 5.58]
all data pooled	charity (hypothetical)	elevation	3375	0.60 [0.51 - 0.68]	1.57 [1.34 - 1.87]
study04	charity (actual)	elevation	195	0.95 [0.34 - 1.56]	1.39 [0.65 - 3.93]
study08	charity (actual)	elevation	416	0.48 [0.04 - 0.90]	0.98 [-0.08 - 4.83]
study09	charity (actual)	elevation	417	0.66 [0.26 - 1.07]	0.55 [0.01 - 1.71]
study10	charity (actual)	elevation	427	0.66 [0.28 - 1.04]	1.02 [0.34 - 2.69]
all data pooled	charity (actual)	elevation	1455	0.64 [0.43 - 0.85]	0.94 [0.55 - 1.53]
study02	friendliness	elevation	252	0.20 [0.04 - 0.35]	0.87 [0.24 - 3.63]
study03	friendliness	elevation	388	0.08 [-0.03 - 0.20]	1.03 [-9.34 - 12.41]
study06	friendliness	elevation	436	0.14 [0.01 - 0.26]	0.55 [-1.06 - 3.91]

study07	friendliness	elevation	375	0.13 [-0.04 - 0.29]	2.34 [-9.56 - 17.47]
study11	friendliness	elevation	122	0.07 [-0.17 - 0.32]	0.29 [-17.57 - 20.96]
study12	friendliness	elevation	108	0.12 [-0.13 - 0.38]	0.40 [-10.69 - 10.32]
all data pooled	friendliness	elevation	1681	0.12 [0.05 - 0.19]	1.29 [0.62 - 3.01]

Note. Summary of study level and pooled data regarding direct behavioral outcomes and elevation as mediator.

Table 2

(S3) Prosocial contagion and mediation thereof by positive affect.

study	behavior	mediator	n	tau [95% CI]	proportion mediated [95 % CI]
study02	charity (hypothetical)	positive affect	494	0.57 [0.34 - 0.80]	0.05 [-0.04 - 0.16]
study03	charity (hypothetical)	positive affect	569	0.39 [0.18 - 0.60]	0.51 [0.27 - 1.08]
study05	charity (hypothetical)	positive affect	456	0.77 [0.55 - 1.00]	0.20 [0.11 - 0.33]
study06	charity (hypothetical)	positive affect	689	0.64 [0.45 - 0.83]	0.22 [0.13 - 0.36]
study07	charity (hypothetical)	positive affect	727	0.59 [0.40 - 0.78]	0.30 [0.19 - 0.48]
study11	charity (hypothetical)	positive affect	184	0.80 [0.42 - 1.17]	0.28 [0.09 - 0.64]
study12	charity (hypothetical)	positive affect	256	0.50 [0.18 - 0.80]	0.29 [0.08 - 0.81]
all data pooled	charity (hypothetical)	positive affect	3375	0.60 [0.52 - 0.68]	0.25 [0.20 - 0.31]
study04	charity (actual)	positive affect	195	0.94 [0.32 - 1.57]	0.30 [0.08 - 0.88]
study08	charity (actual)	positive affect	416	0.48 [0.10 - 0.88]	0.14 [-0.13 - 0.79]
study09	charity (actual)	positive affect	417	0.67 [0.27 - 1.07]	0.05 [-0.06 - 0.24]
study10	charity (actual)	positive affect	427	0.66 [0.27 - 1.06]	0.26 [0.05 - 0.69]
all data pooled	charity (actual)	positive affect	1455	0.64 [0.42 - 0.86]	0.16 [0.06 - 0.30]
study02	friendliness	positive affect	252	0.20 [0.05 - 0.35]	0.04 [-0.08 - 0.26]
study03	friendliness	positive affect	388	0.08 [-0.03 - 0.19]	0.24 [-2.28 - 3.22]
study06	friendliness	positive affect	436	0.14 [0.02 - 0.26]	0.08 [-0.22 - 0.67]

study07	friendliness	positive affect	375	0.14 [-0.02 - 0.31]	0.34 [-1.36 - 2.70]
study11	friendliness	positive affect	122	0.06 [-0.18 - 0.31]	0.04 [-4.29 - 4.47]
study12	friendliness	positive affect	108	0.12 [-0.16 - 0.37]	0.02 [-1.34 - 1.45]
all data pooled	friendliness	positive affect	1681	0.12 [0.05 - 0.19]	0.22 [0.09 - 0.54]

Note. Summary of study level and pooled data regarding direct behavioral outcomes and positive affect as mediator.

Table 3

(S4) Prosocial contagion and mediation thereof by prosocial motives.

study	behavior	mediator	n	tau [95% CI]	proportion mediated [95 % CI]
study02	charity (hypothetical)	prosocial motives	494	0.57 [0.34 - 0.80]	0.96 [0.65 - 1.57]
study03	charity (hypothetical)	prosocial motives	569	0.39 [0.19 - 0.59]	2.71 [1.64 - 5.48]
study05	charity (hypothetical)	prosocial motives	456	0.78 [0.55 - 1.00]	1.25 [0.81 - 1.93]
study06	charity (hypothetical)	prosocial motives	689	0.64 [0.45 - 0.83]	1.40 [0.94 - 2.09]
study07	charity (hypothetical)	prosocial motives	727	0.59 [0.39 - 0.79]	1.91 [1.37 - 2.83]
study11	charity (hypothetical)	prosocial motives	184	0.79 [0.42 - 1.18]	1.63 [0.85 - 3.17]
study12	charity (hypothetical)	prosocial motives	256	0.51 [0.19 - 0.84]	2.21 [1.17 - 5.72]
all data pooled	charity (hypothetical)	prosocial motives	3375	0.60 [0.51 - 0.68]	1.47 [1.24 - 1.76]
study04	charity (actual)	prosocial motives	195	0.95 [0.35 - 1.58]	1.42 [0.64 - 4.25]
study08	charity (actual)	prosocial motives	416	0.49 [0.09 - 0.90]	1.23 [0.19 - 5.29]
study09	charity (actual)	prosocial motives	417	0.66 [0.28 - 1.07]	0.53 [-0.10 - 1.70]
study10	charity (actual)	prosocial motives	427	0.66 [0.28 - 1.05]	1.02 [0.23 - 2.72]
all data pooled	charity (actual)	prosocial motives	1455	0.64 [0.43 - 0.86]	0.99 [0.54 - 1.66]
study02	friendliness	prosocial motives	252	0.20 [0.05 - 0.36]	0.43 [0.02 - 1.83]
study03	friendliness	prosocial motives	388	0.08 [-0.03 - 0.19]	0.83 [-6.81 - 12.48]
study06	friendliness	prosocial motives	436	0.14 [0.02 - 0.26]	0.57 [-1.19 - 4.03]

study07	friendliness	prosocial motives	375	0.13 [-0.04 - 0.31]	2.13 [-11.70 - 19.70]
study11	friendliness	prosocial motives	122	0.06 [-0.18 - 0.30]	0.11 [-13.71 - 17.32]
study12	friendliness	prosocial motives	108	0.12 [-0.14 - 0.38]	0.26 [-11.60 - 13.14]
all data pooled	friendliness	prosocial motives	1681	0.12 [0.05 - 0.19]	0.80 [0.18 - 2.11]

Note. Summary of study level and pooled data regarding direct behavioral outcomes and prosocial motives subscale of elevation measure as mediator.

Table 4

(S5) Prosocial contagion and mediation thereof by folk affect terms subscale.

study	behavior	mediator	n	tau [95% CI]	proportion mediated [95 % CI]
study02	charity (hypothetical)	folk affect	494	0.57 [0.35 - 0.80]	1.07 [0.69 - 1.80]
study03	charity (hypothetical)	folk affect	569	0.39 [0.18 - 0.60]	2.32 [1.40 - 5.18]
study05	charity (hypothetical)	folk affect	456	0.78 [0.55 - 1.00]	1.08 [0.72 - 1.63]
study06	charity (hypothetical)	folk affect	689	0.64 [0.44 - 0.83]	1.17 [0.77 - 1.79]
study07	charity (hypothetical)	folk affect	727	0.59 [0.41 - 0.79]	1.75 [1.24 - 2.58]
study11	charity (hypothetical)	folk affect	184	0.79 [0.42 - 1.17]	1.76 [0.97 - 3.62]
study12	charity (hypothetical)	folk affect	256	0.51 [0.17 - 0.84]	1.61 [0.83 - 4.49]
all data pooled	charity (hypothetical)	folk affect	3375	0.60 [0.51 - 0.68]	1.40 [1.19 - 1.68]
study04	charity (actual)	folk affect	195	0.95 [0.36 - 1.55]	1.44 [0.67 - 4.00]
study08	charity (actual)	folk affect	416	0.49 [0.09 - 0.89]	0.93 [-0.02 - 4.25]
study09	charity (actual)	folk affect	417	0.67 [0.28 - 1.05]	0.42 [-0.06 - 1.35]
study10	charity (actual)	folk affect	427	0.67 [0.26 - 1.05]	0.88 [0.22 - 2.50]
all data pooled	charity (actual)	folk affect	1455	0.65 [0.44 - 0.86]	0.86 [0.48 - 1.45]
study02	friendliness	folk affect	252	0.20 [0.06 - 0.35]	0.92 [0.25 - 3.21]
study03	friendliness	folk affect	388	0.08 [-0.03 - 0.19]	0.85 [-5.78 - 10.33]
study06	friendliness	folk affect	436	0.14 [0.02 - 0.25]	0.65 [-0.87 - 4.01]

study07	friendliness	folk affect	375	0.13 [-0.03 - 0.30]	2.52 [-23.62 - 25.71]
study11	friendliness	folk affect	122	0.06 [-0.19 - 0.31]	0.52 [-15.92 - 20.47]
study12	friendliness	folk affect	108	0.12 [-0.15 - 0.39]	0.10 [-9.21 - 9.03]
all data pooled	friendliness	folk affect	1681	0.12 [0.05 - 0.19]	1.34 [0.64 - 3.23]

Note. Summary of study level and pooled data regarding behavioral direct outcomes and folk affect subscale of elevation as mediator.

Table 5

(S6) Prosocial contagion and mediation thereof by the somatic subscale.

study	behavior	mediator	n	tau [95% CI]	proportion mediated [95 % CI]
study02	charity (hypothetical)	somatic	494	0.56 [0.35 - 0.79]	0.83 [0.48 - 1.44]
study03	charity (hypothetical)	somatic	569	0.39 [0.18 - 0.58]	1.40 [0.84 - 2.99]
study05	charity (hypothetical)	somatic	456	0.78 [0.55 - 0.99]	0.56 [0.32 - 0.90]
study06	charity (hypothetical)	somatic	689	0.64 [0.44 - 0.83]	0.73 [0.48 - 1.11]
study07	charity (hypothetical)	somatic	727	0.60 [0.41 - 0.79]	0.95 [0.69 - 1.40]
study11	charity (hypothetical)	somatic	184	0.79 [0.42 - 1.17]	0.91 [0.41 - 1.82]
study12	charity (hypothetical)	somatic	256	0.50 [0.17 - 0.83]	1.03 [0.52 - 3.04]
all data pooled	charity (hypothetical)	somatic	3375	0.60 [0.52 - 0.69]	0.86 [0.72 - 1.03]
study04	charity (actual)	somatic	195	0.93 [0.33 - 1.51]	0.43 [0.02 - 1.48]
study08	charity (actual)	somatic	416	0.48 [0.06 - 0.89]	0.18 [-0.41 - 1.22]
study09	charity (actual)	somatic	417	0.67 [0.27 - 1.07]	0.36 [0.04 - 1.02]
study10	charity (actual)	somatic	427	0.66 [0.28 - 1.04]	0.43 [0.07 - 1.20]
all data pooled	charity (actual)	somatic	1455	0.64 [0.43 - 0.85]	0.34 [0.15 - 0.63]
study02	friendliness	somatic	252	0.20 [0.06 - 0.35]	0.74 [0.07 - 2.61]
study03	friendliness	somatic	388	0.08 [-0.02 - 0.19]	0.83 [-9.10 - 10.38]
study06	friendliness	somatic	436	0.14 [0.01 - 0.26]	0.15 [-0.95 - 1.60]

study07	friendliness	somatic	375	0.13 [-0.03 - 0.29]	1.02 [-5.70 - 10.05]
study11	friendliness	somatic	122	0.07 [-0.19 - 0.32]	0.08 [-10.11 - 12.22]
study12	friendliness	somatic	108	0.11 [-0.16 - 0.37]	0.49 [-5.96 - 8.15]
all data pooled	friendliness	somatic	1681	0.12 [0.06 - 0.19]	0.75 [0.32 - 1.67]

Note. Summary of study level and pooled data regarding charity as outcome and the somatic subscale of the elevation measure as mediator.

Study level versions of main text behavior summary figure

Figures S1 displays study-level versions of the main text summary figure.

Pledged versus actual charitable donation

Figures S2 validates that charity pledges were generally consistent with actual donations.

Study level versions of main text emotion and attitude summary plots

Figures S3 to S13 present study-level versions of the constituents of main text Figure 2.

Contrasting the attitudes and emotions of the two study populations

There are slight differences in emotion and attitude between our two study populations; Angelinos who participated in the field report lower levels of emotion in response to the Prosocial video and less idealistic attitudes than do MTurk workers (Figure S14). One possible reason for reduced emotional response is that the field setting included many more distractions than the environment experienced by typical MTurk workers.

Sex differences in attitude and emotion

In our data, women report slightly higher levels of emotion and idealistic attitude (Figure S15).

Framing of emotion scale items

In Studies 1 and 2, emotion measures (elevation scale and positive affect items) were presented as “right now I feel [item].” Study 1 constrasted the Prosocial video condition with a no video control condition; Study 2 used the Athletic video control condition. All other studies presented such items as “the video made me feel [item].” Our concern with the former framing is that the question can be interpretted as a response to the entire experience of

participating in a study, rather than specifically a reaction to the video stimuli. This concern can be expected to manifest differently for the different subscales of the elevation measure.

For the prosocial motives items framed as “right now I feel”, our MTurk participants–workers who accepted a job–are essentially being asked if they feel like helping. The Prosocial video can be expected to enhance such feelings, but, since all participants essentially agreed to help researchers by completing the study, we should expect fairly high levels on this scale even in control conditions. Further, since idealism indexes baseline expectations and valuations of cooperation, we should expect general feelings of cooperative intent to be correlated with idealism in these control conditions. Figure S16 shows exactly this relationship, and shows that this changes drastically when motives are specifically attributed to the stimuli.

Similarly, folk affect terms such as “inspired” and “moved” could be used to describe the experience of participating in a scientific study and/or watching an Athletic video, and the tendency to endorse such descriptions might also be positively correlated with idealism. As mentioned in main text, this effect is somewhat present when the measure is specific to the stimuli; Figure S17 suggests this trend may be more severe when scale items framed more generally.

Unlike the previous two subscales, the somatic subscale seems less likely to be affected by framing–it is intuitively implausible that participating in a mildly enjoyable research study or watching an Athletic video would regularly induce moderate levels of goosebumps, for example. Indeed, Figure S18 suggests that framing is unimportant for the somatic subscale.

Finally, generalized positive affect is not strongly affected by framing or condition (Figure S19)

Scale reliabilities

The idealism scale typically has alphas > 0.85, with some field studies and/or earlier versions of scales slightly lower. Elevation scale alphas are >.95, with subscales and Positive Affect scale usually >.80. For details, see Table 6.

Table 6

Scale alphas

study	idealism	elevation	somatic	folk_affect	prosocial_motives	positive_affect
study01	0.93	0.97	0.91	0.96	0.90	0.74
study02	0.93	0.96	0.88	0.94	0.92	0.74
study03	*	0.97	0.88	0.96	0.96	0.81
study04	0.84	0.97	0.86	0.95	0.94	0.82
study05	0.89	0.98	0.89	0.96	0.97	0.81
study06	0.87	0.97	0.88	0.95	0.96	0.82
study07	0.88	0.97	0.91	0.96	0.96	0.83
study08	0.73	0.96	0.80	0.93	0.92	0.80
study09	0.86	0.96	0.84	0.94	0.92	0.81
study10	0.77	0.96	0.83	0.95	0.94	0.80
study11	0.91	0.97	0.88	0.95	0.97	0.81
study12	0.87	0.97	0.87	0.96	0.95	0.87
study13	0.93	0.97	0.88	0.96	0.95	0.86
study14	0.93	0.96	0.88	0.96	0.93	0.83
study15	0.82	0.95	0.85	0.94	0.91	0.83

Note. * - Two different versions of the idealism scale were used in Study 3. Their alphas were 0.92 and 0.91.

Figures

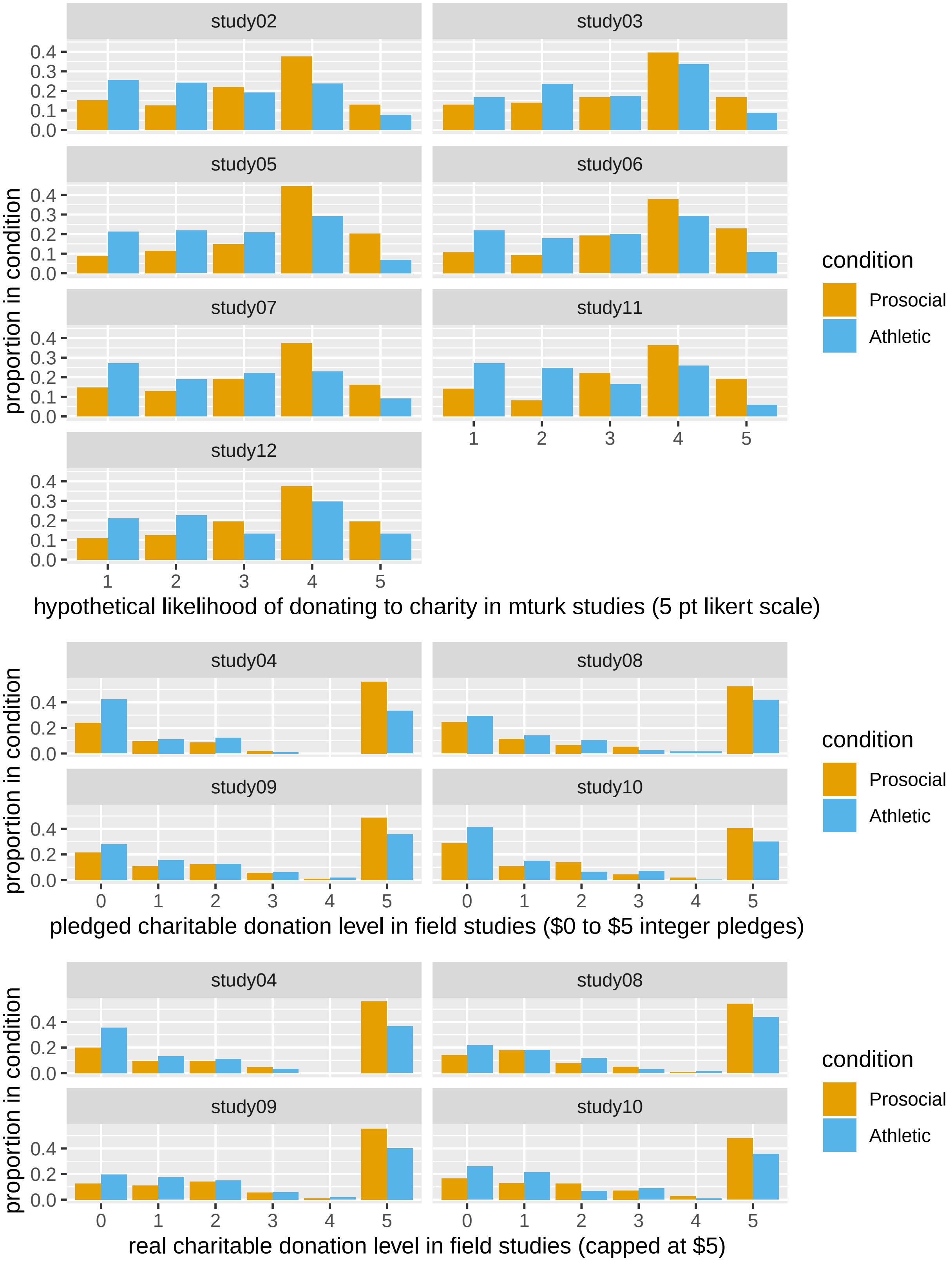

Figure 1. (S1) Study level versions of main text behavior plot, including charity pledges from field studies.

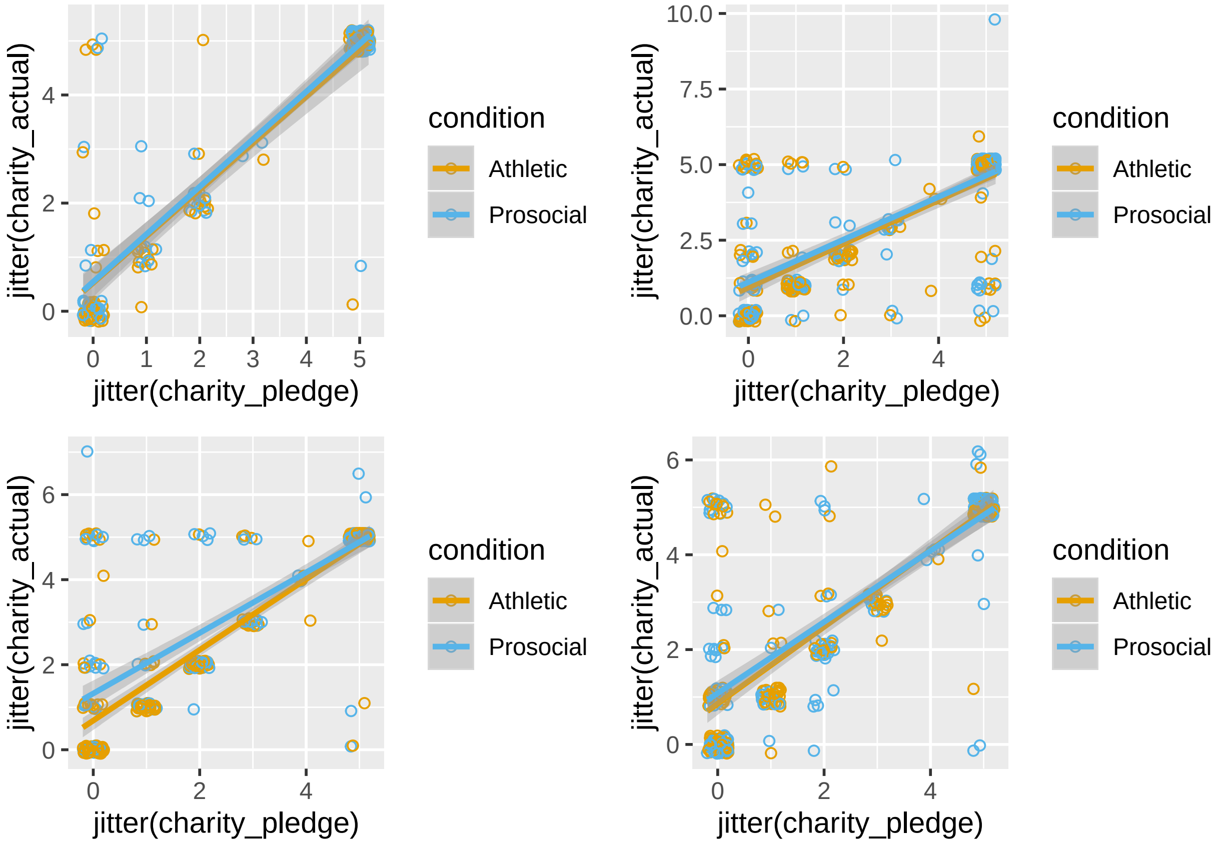

Figure 2. (S2) Pledged vs actual donations in field studies. This is a diagnostic tool to validate that these two measures are fairly closely related. Participants sometimes gave more than their $5 participation fee, which was not a pledge option.

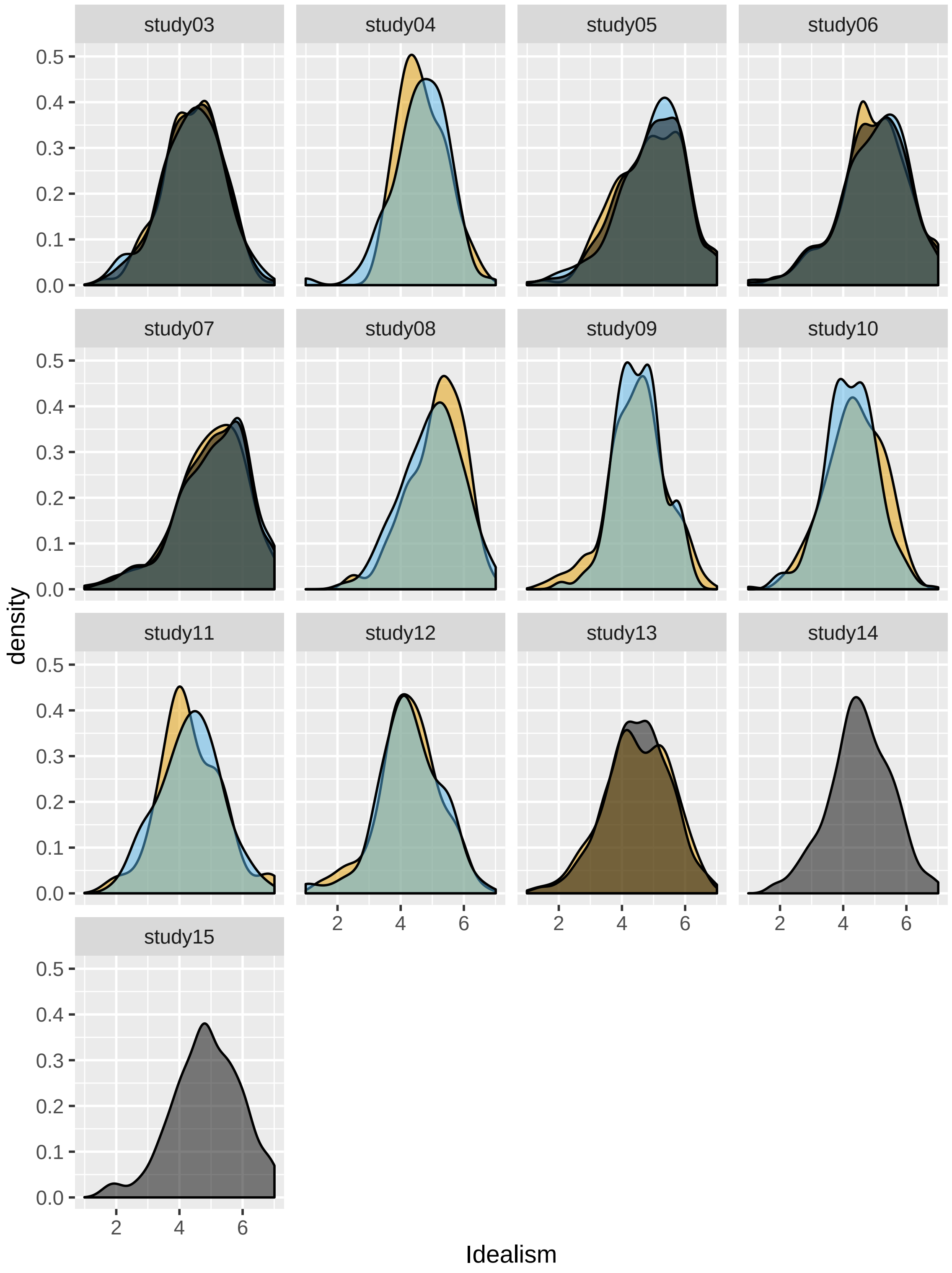

Figure 3. (S3) Study level versions of main text idealism density plot.

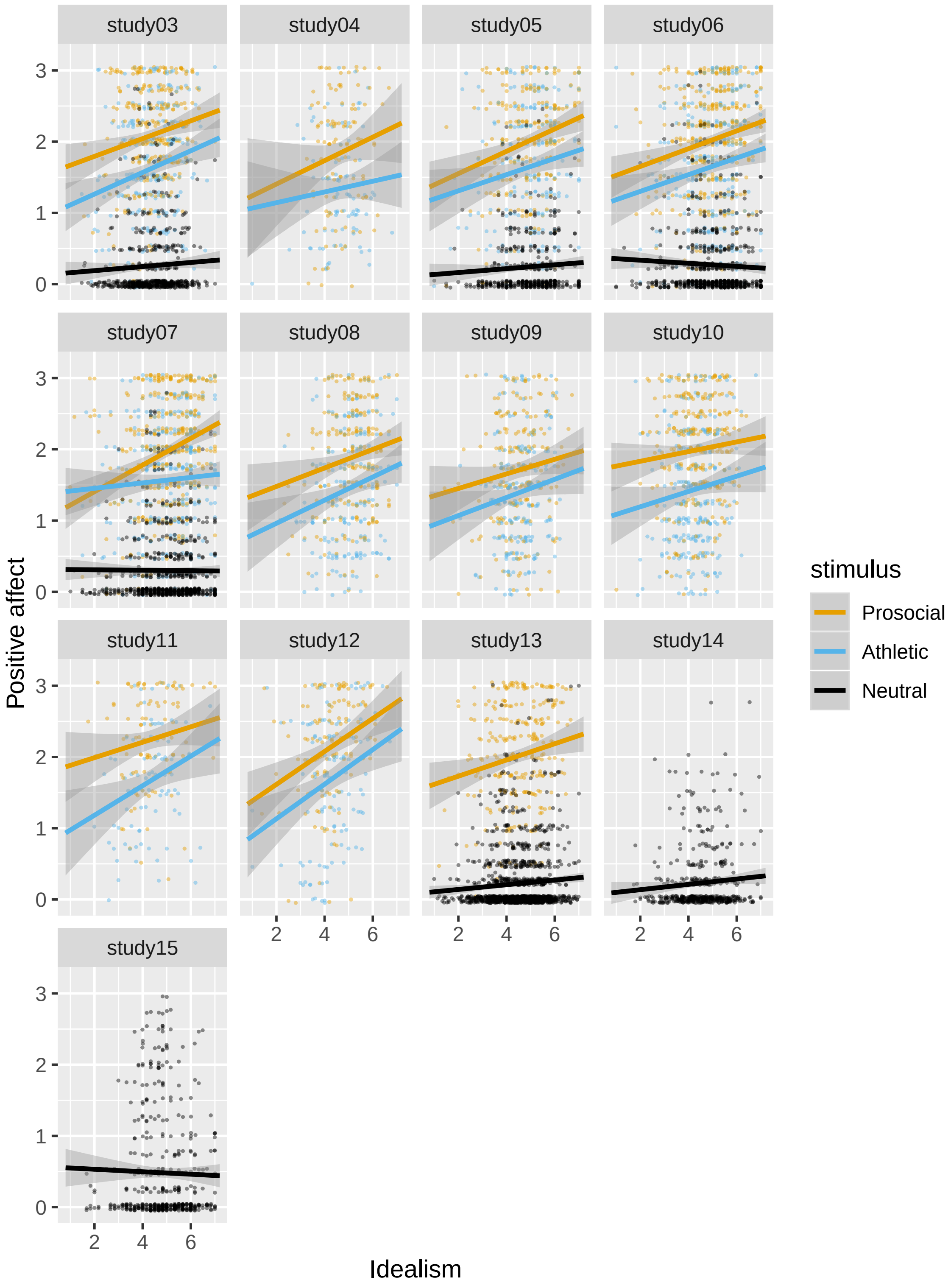

Figure 4. (S4) Study level versions of main text scatter plot for positive affect.

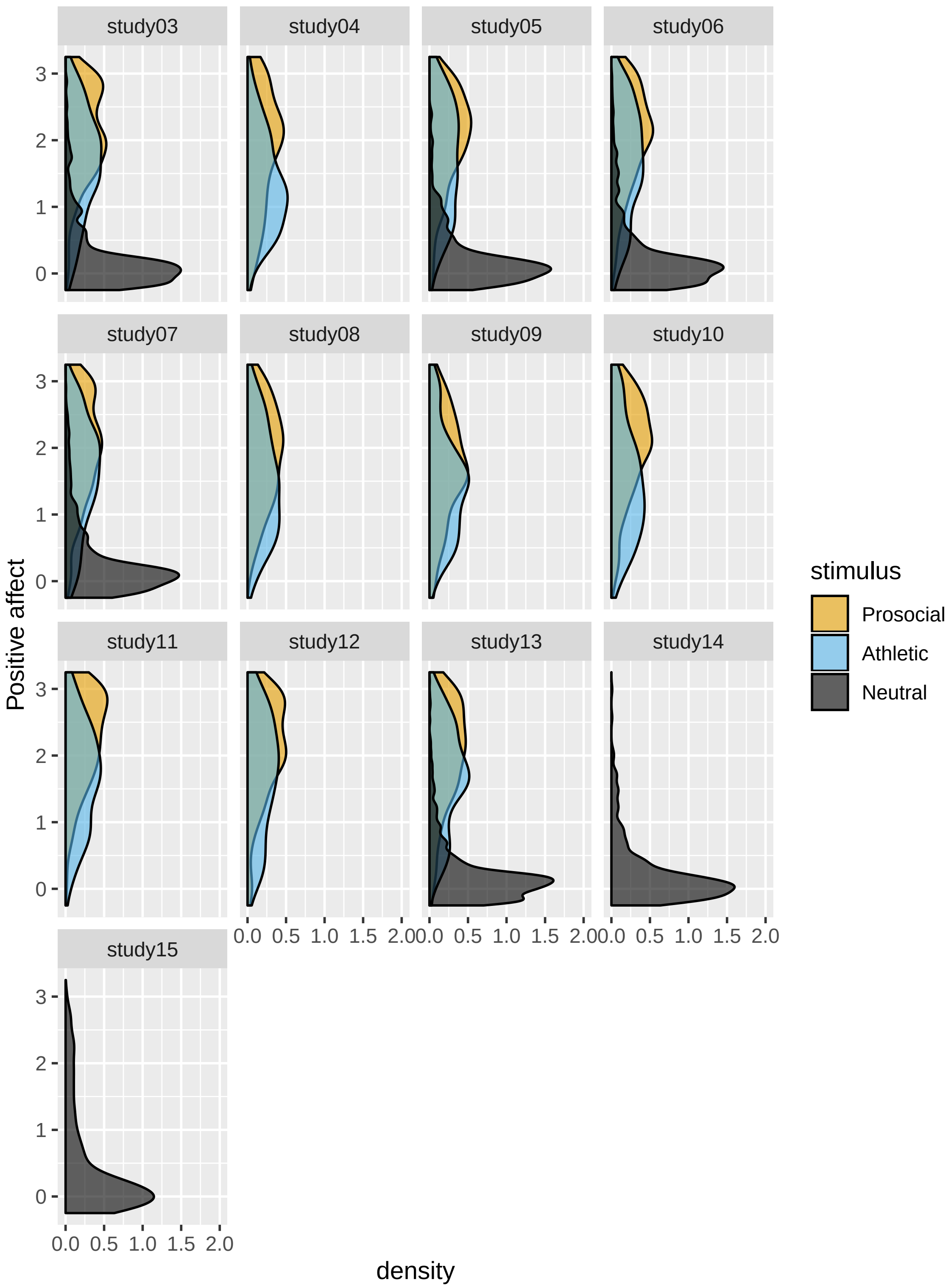

Figure 5. (S5) Study level versions of main text emotion density plots for positive affect.

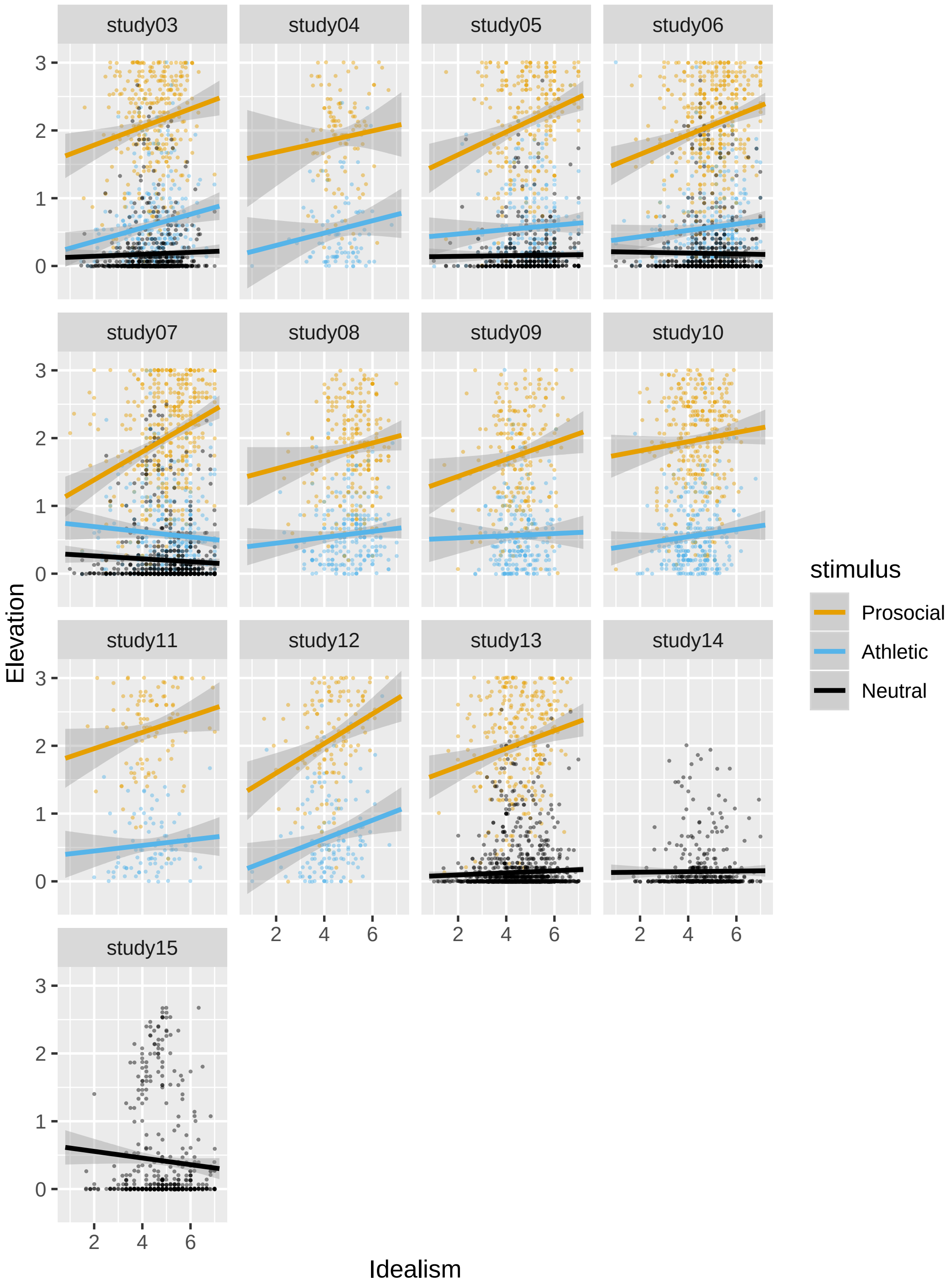

Figure 6. (S6) Study level versions of main text scatter plots for elevation.

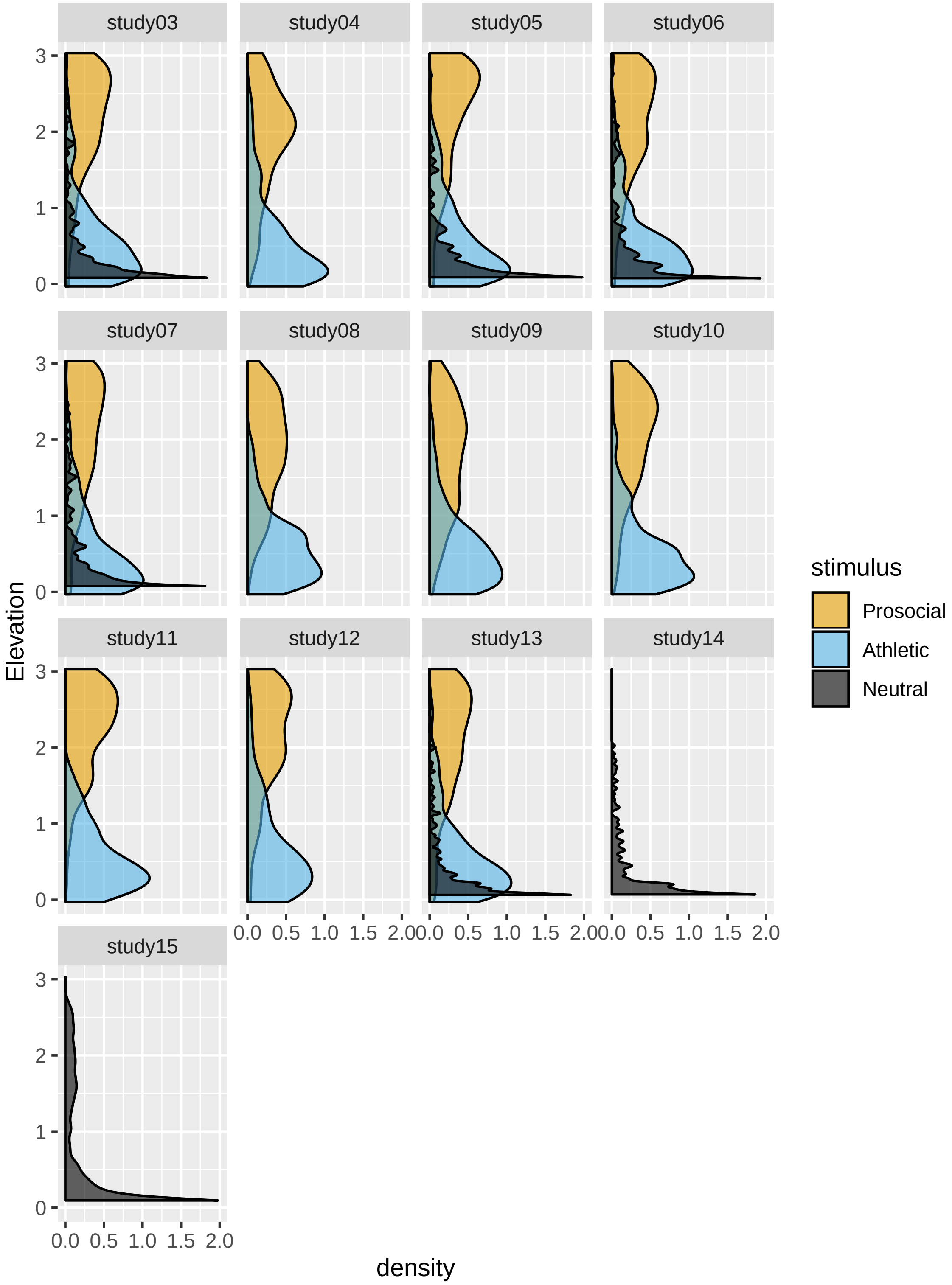

Figure 7. (S7) Study level version of main text emotion density plots for elevation.

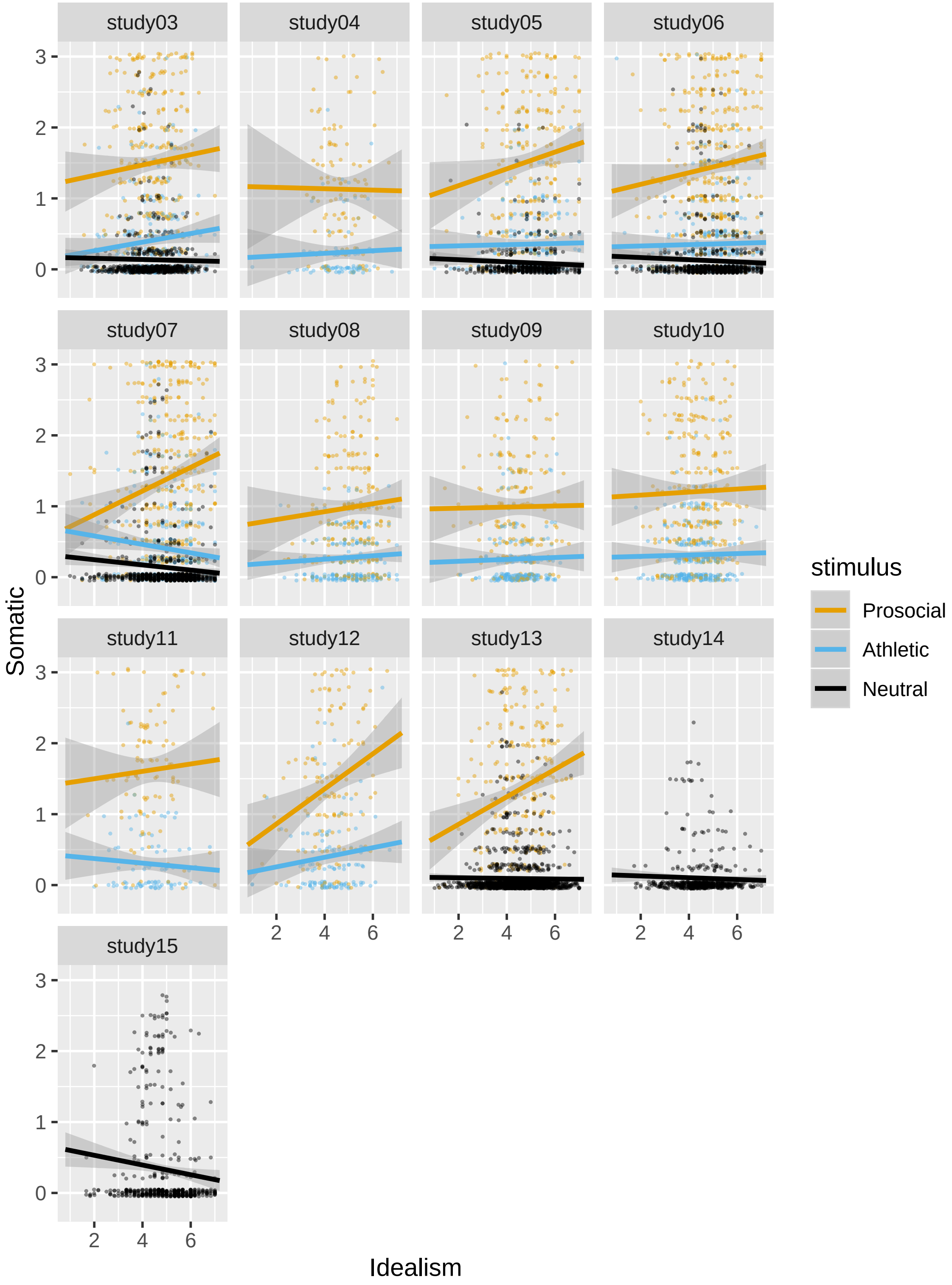

Figure 8. (S8) Study level versions of main text scatter plots for the somatic subscale of elevation.

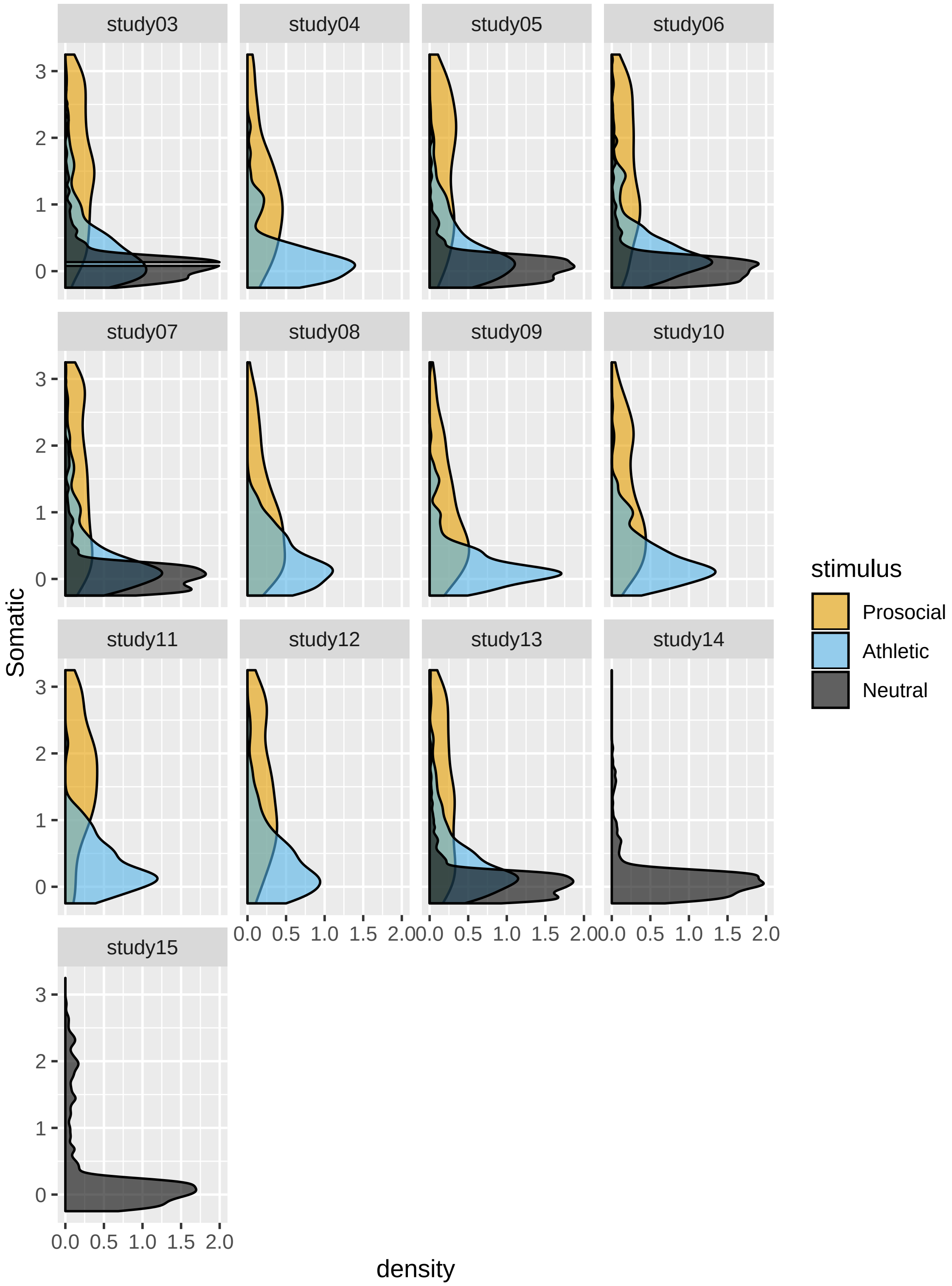

Figure 9. (S9) Study level version of main text emotion density plots for the somatic subscale of elevation.

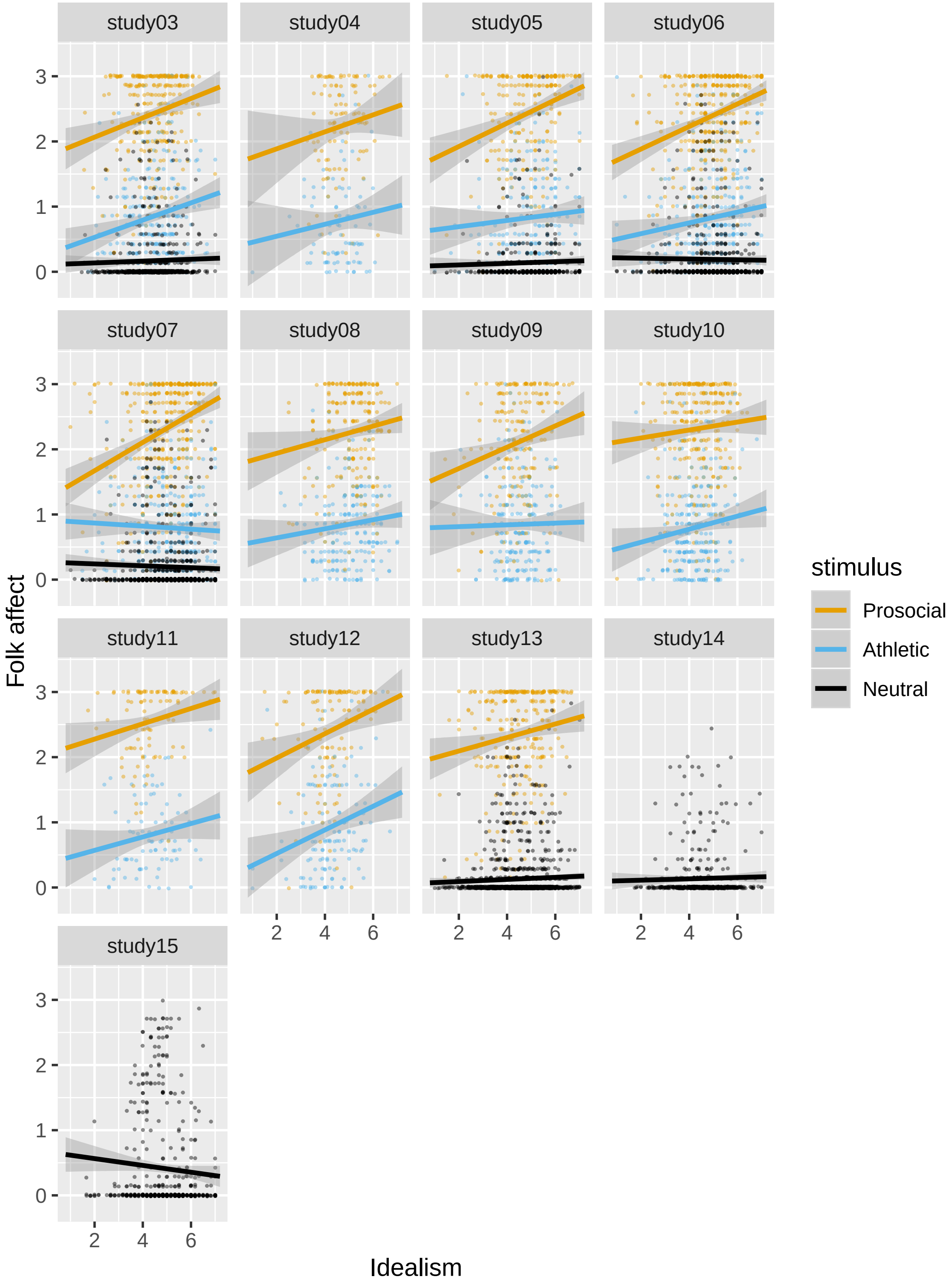

Figure 10. (S10) Study level versions of main text scatter plots for the folk affect terms subscale of elevation.

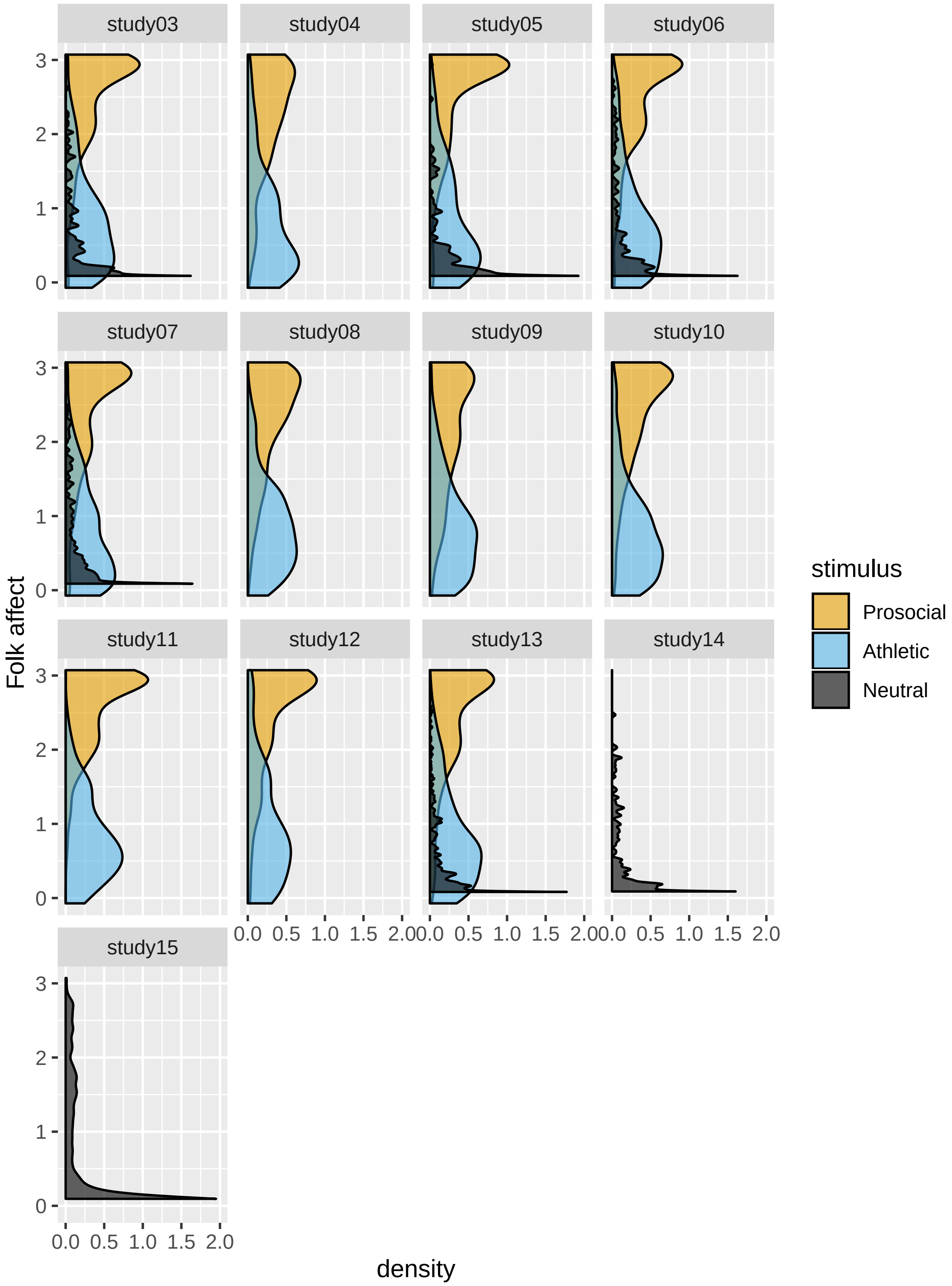

Figure 11. (S11) Study level version of main text emotion density plots for the folk affect terms subscale of elevation.

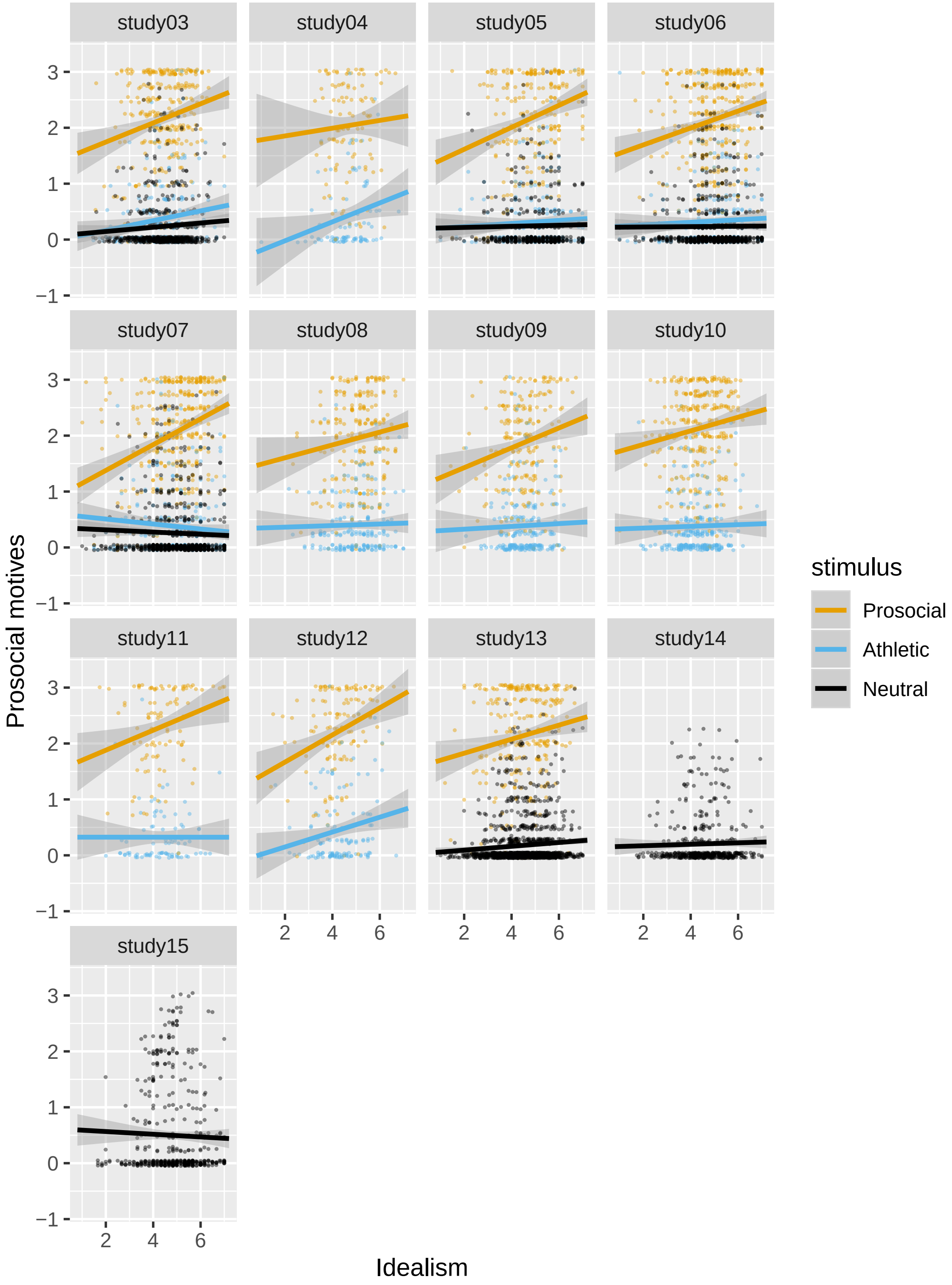

Figure 12. (S12) Study level versions of main text scatter plots for the prosocial motives subscale of elevation.

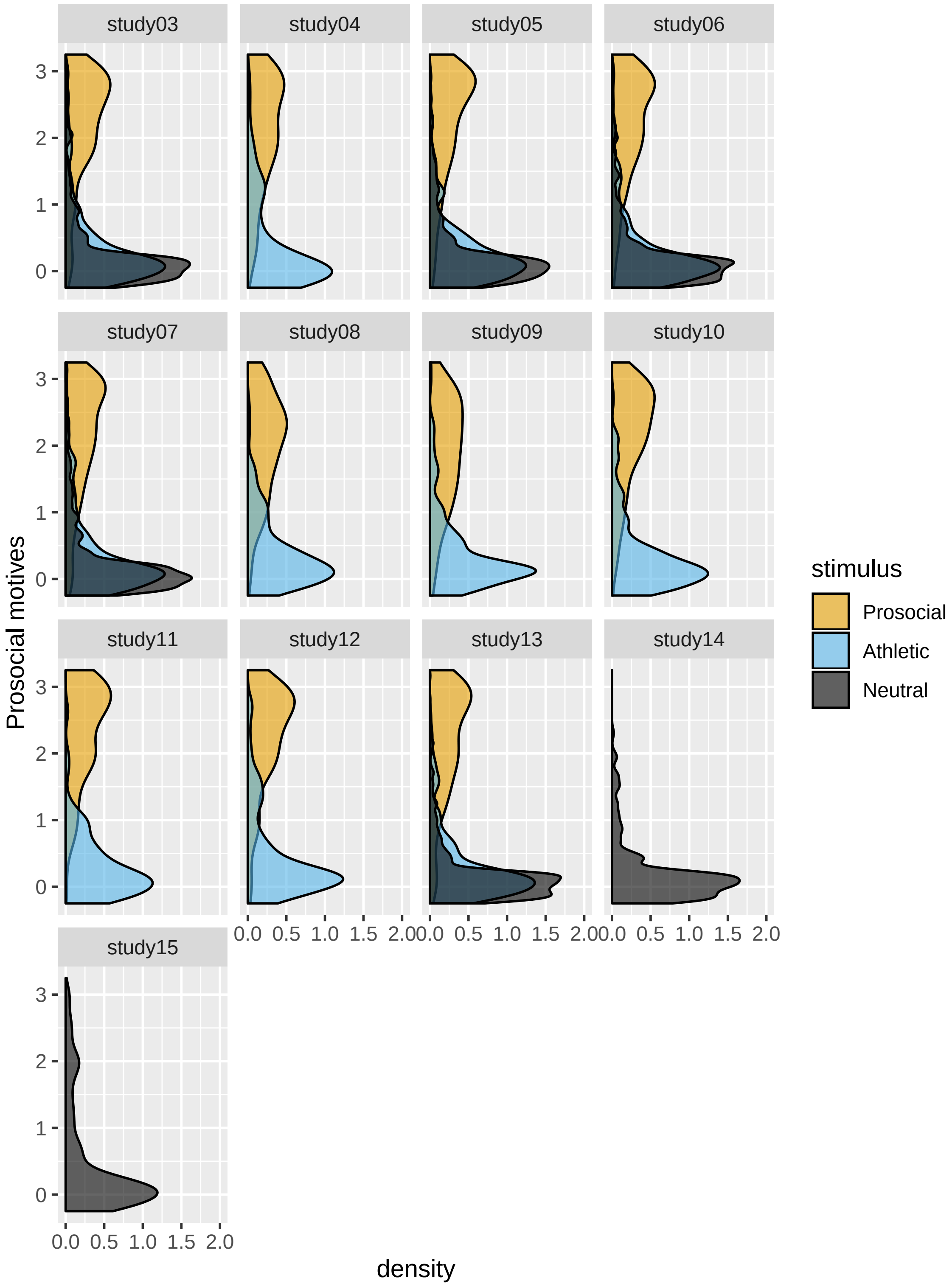

Figure 13. (S13) Study level version of main text emotion density plots for the prosocial motives subscale of elevation.

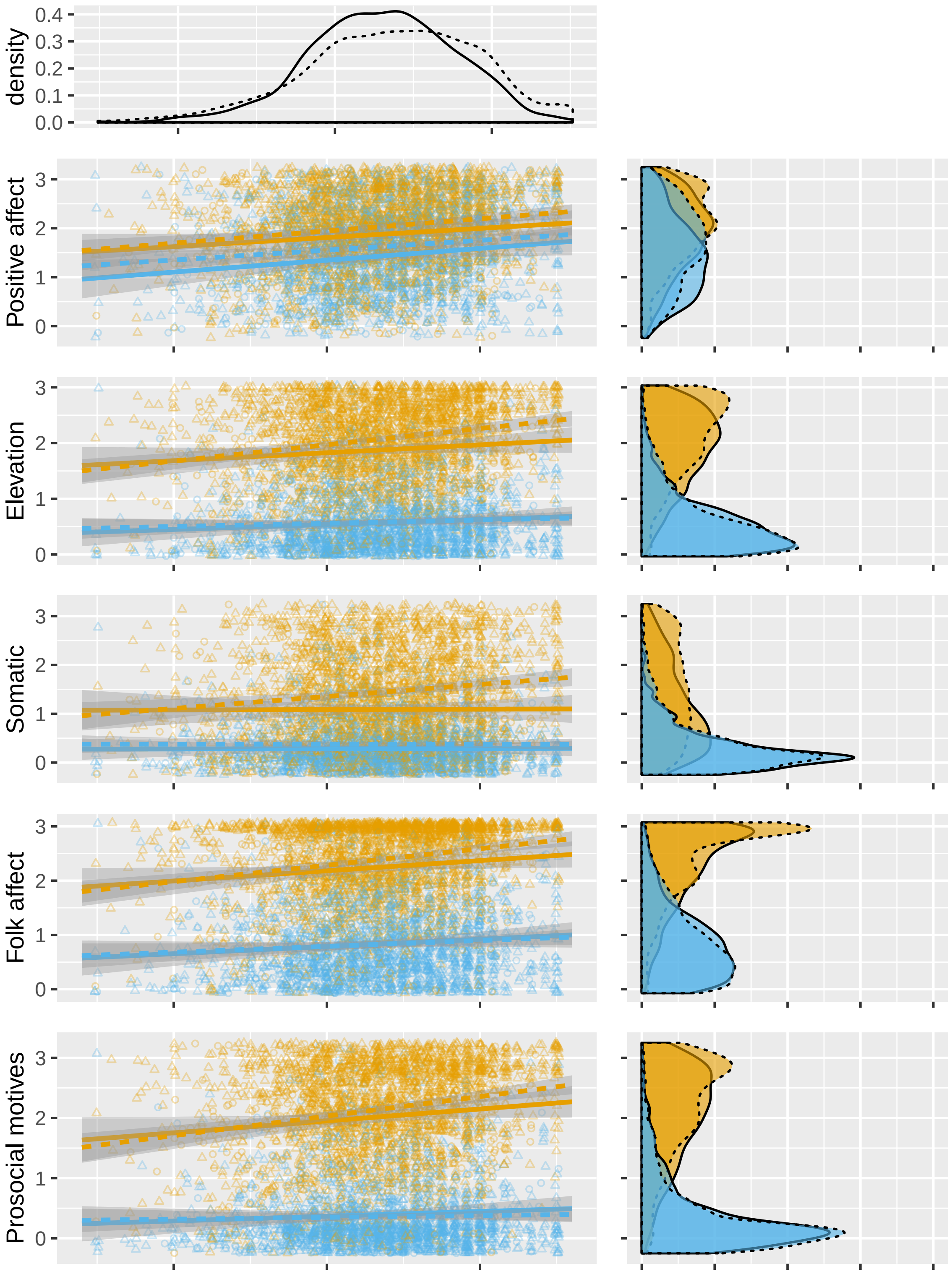

Figure 14. (S14) Contrasting MTurk populations in online studies (dotted lines) and Angelinos in field studies (solid). Emotional response to Prosocial video is somewhat muted in the field studies.

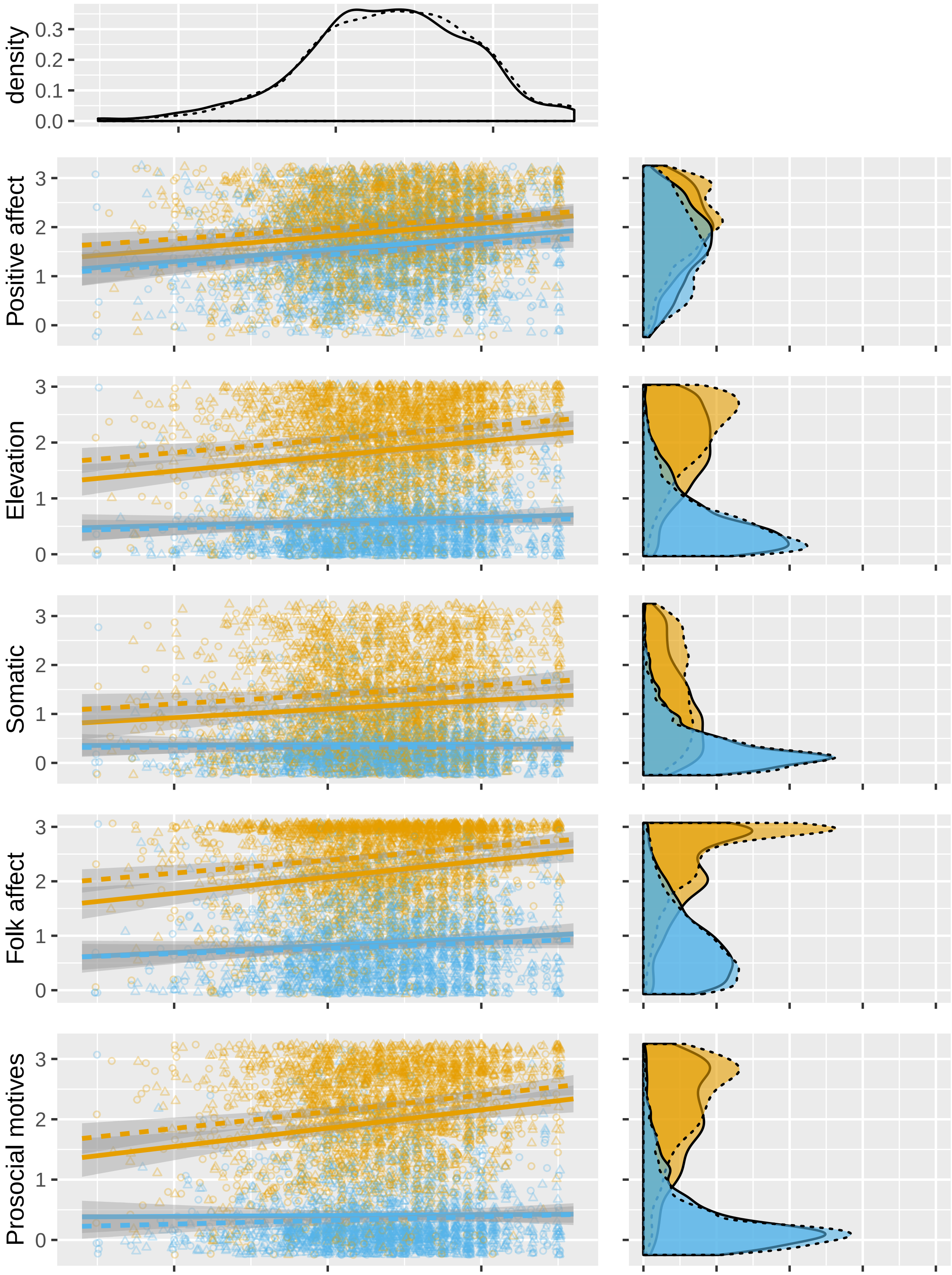

Figure 15. (S15) Sex Differences. Women (dotted lines) report higher levels of emotion and idealism than men (solid).

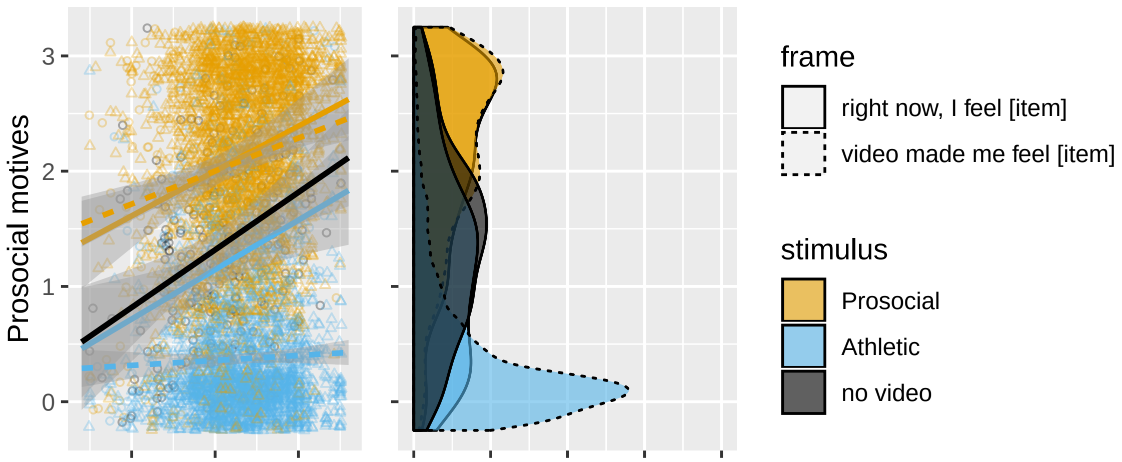

Figure 16. (S16) Prosocial motives subscale of elevation when framed as "right now I feel" (Studies 1 and 2) versus "the video made me feel" (all other studies)

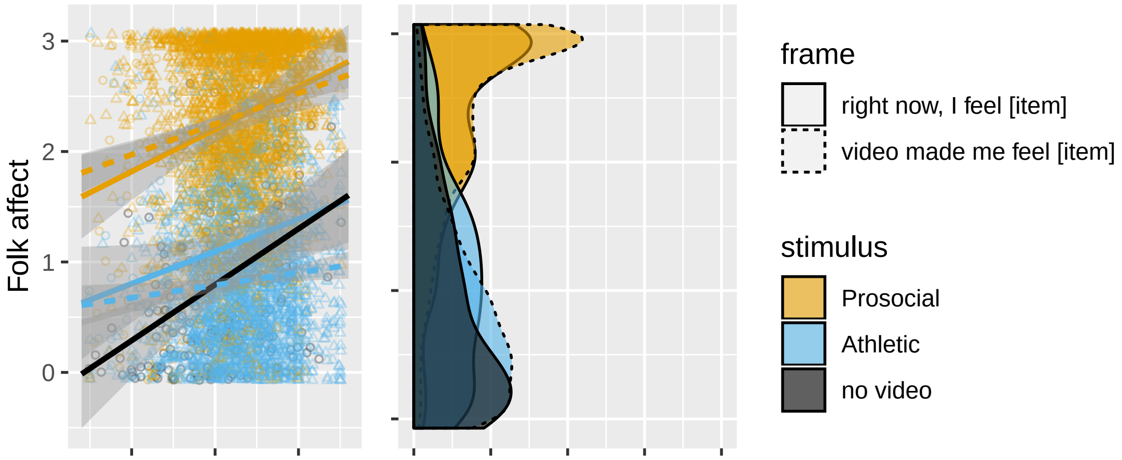

Figure 17. (S17) Folk affect terms subscale of elevation when framed as "right now I feel" (Studies 1 and 2) versus "the video made me feel" (all other studies)

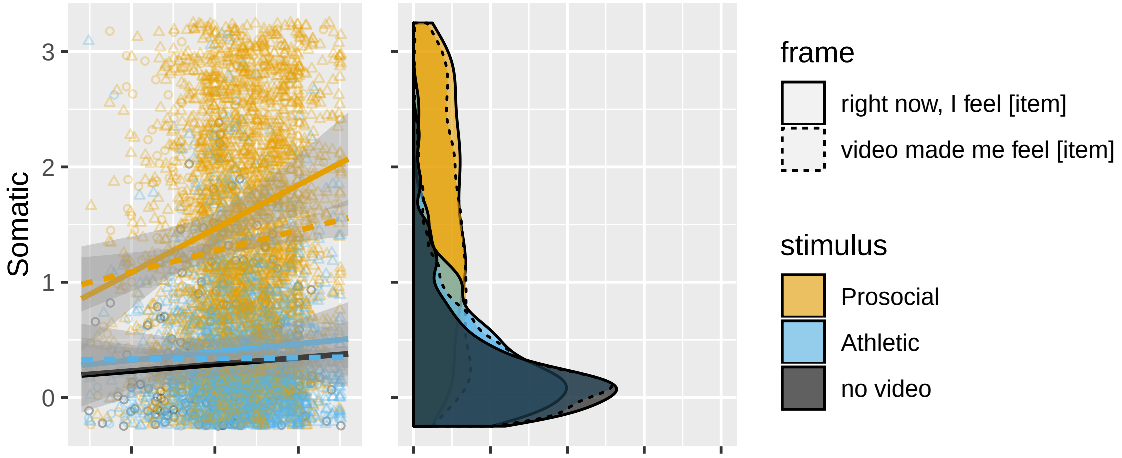

Figure 18. (S18) Somatic subscale of elevation when framed as "right now I feel" (Studies 1 and 2) versus "the video made me feel" (all other studies)

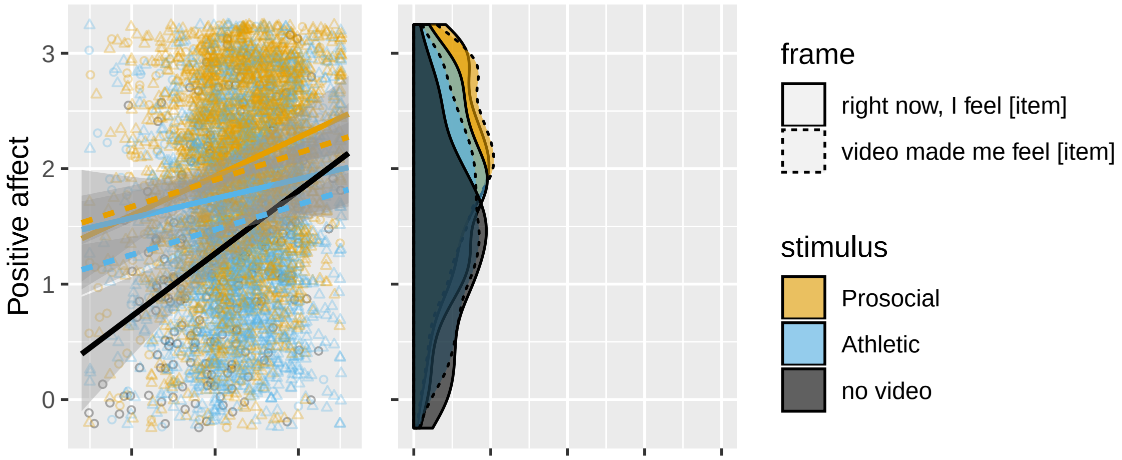

Figure 19. (S19) Positive affect when framed as "right now I feel" (Studies 1 and 2) versus "the video made me feel" (all other studies)
